# Average and Local Structure of La_1–*x*_Sr*_*x*_*Fe_1–*y*_Mn*_*y*_*O_3−δ_ Chemical Looping Oxygen Carrier Materials

**DOI:** 10.1021/acs.chemmater.5c00388

**Published:** 2025-05-02

**Authors:** Daniel M. Telford, Wenting Hu, Ian S. Metcalfe, Martin O. Jones, Paul F. Henry, John S. O. Evans

**Affiliations:** † School of Engineering, Newcastle University, Newcastle-upon-Tyne NE1 7RU, U.K.; ‡ ISIS Neutron and Muon Source, Science and Technology Facilities Council, Rutherford Appleton Laboratory, Didcot OX11 0QX, U.K.; § Department of Chemistry, Durham University, Durham DH1 3LE, U.K.

## Abstract

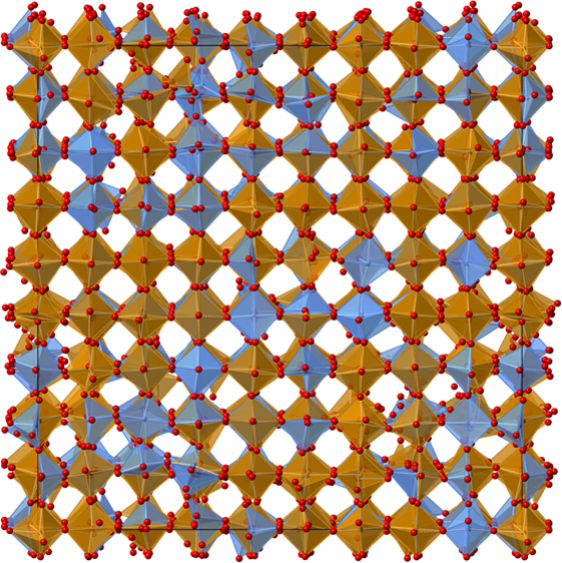

Nonstoichiometric mixed-metal oxides in the La_1–*x*_Sr*_*x*_*Fe_1–*y*_Mn*_*y*_*O_3__–δ_ family are promising oxygen carrier materials for chemical looping processes, including clean hydrogen production from the water-gas shift reaction. The crystal structure variation of these materials during redox reactions is key to the performance of a chemical looping system. Pair distribution function analysis of neutron total scattering data has provided new insight into the local structure of these materials before and after reduction to their working states. Comparison with experimental data for structurally related vacancy-ordered SrFeO_3__–δ_ compounds (Sr_8_Fe_8_O_23_, Sr_4_Fe_4_O_11_, and Sr_2_Fe_2_O_5_) allows direct qualitative insight into local B-site coordination environments. A big-box modeling approach incorporating magnetic contributions to the Bragg data on supercells with A- and B-site disorder and mixed B-site coordination gives quantitative information on local structural distortions and coordination polyhedra. For the Mn-doped materials, this modeling shows that Mn has a higher oxidation state than Fe in oxidized samples. Magnetic structures of all ordered compounds have been determined from neutron powder diffraction data, and variable-temperature studies of La_0.6_Sr_0.4_FeO_3_, La_0.6_Sr_0.4_FeO_2.8_, La_0.6_Sr_0.4_Fe_0.67_Mn_0.33_O_3_, and La_0.6_Sr_0.4_Fe_0.67_Mn_0.33_O_2.8_ have been used to determine magnetic ordering temperatures.

## 1 Introduction

**Figure 1:**
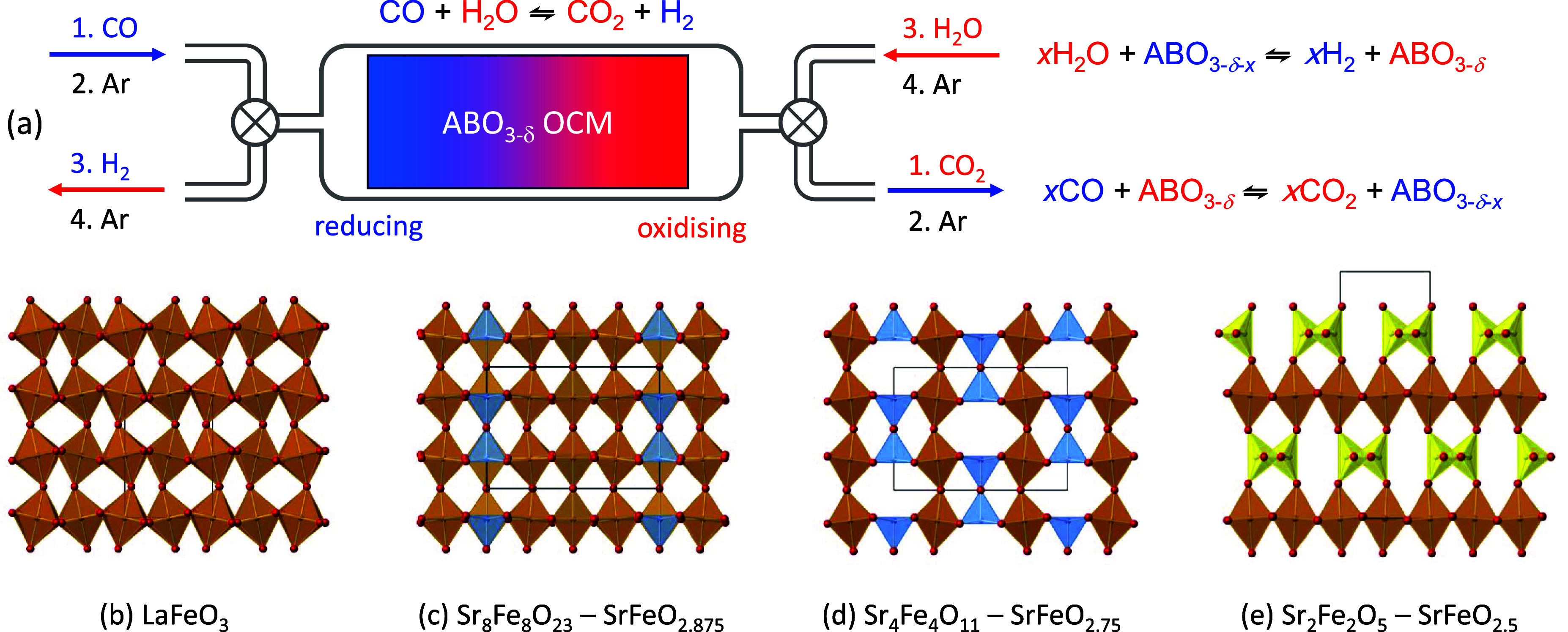
(a) Schematic of a chemical looping memory reactor using a perovskite oxygen carrier material. Under counterflow of CO then H_2_O (each followed by an Ar purge), an oxygen chemical potential develops along an ABO_3−δ_ bed. High conversions to CO_2_ and H_2_ then give rise to separated streams of product gases. (b–e) Structures of AFeO_3−δ_ perovskite derivatives with ordered vacancy structures. FeO_6/2_ octahedra shown in brown, FeO_5/2_ square pyramids in blue, and FeO_4/2_ tetrahedra in yellow. A-site cations omitted for clarity.

Chemical looping (CL) is a process in which an overall chemical reaction is broken down into two or more separate stages. It offers a number of advantages over conventional mixed reactions.^1−9^ One recent breakthrough has described how CL can overcome normal reaction equilibrium limitations when a nonstoichiometric oxide (ABO_3−δ_) is used as an oxygen carrier material (OCM) in a “memory reactor”.^10−12^ For example, by splitting the conventional, homogeneous water-gas shift reaction (WGS; CO + H_2_O ⇋ CO_2_ + H_2_) into two consecutive heterogeneous reactions (water reduction and carbon monoxide oxidation, see Figure 1) overall conversions far in excess of the equilibrium-limited 50% (at 820 °C) can be achieved. This is done by cyclical operation of the reactor with a counterflow of the two reactant gases that generates a smooth gradient of oxygen content, and therefore oxygen chemical potential, along an ABO_3−δ_ bed. This gradient means that the CO flow exits at the highly oxidizing end of the bed and undergoes high conversion to CO_2_, while the H_2_O flow exits at the reducing end and undergoes high conversion to H_2_.^12−14^ Overall, WGS conversions approaching 100% have been demonstrated using this method over thousands of reactor cycles.

In order to understand and optimize the properties of perovskite OCMs, it is important to know their average and local structure and the oxygen content under operating conditions.^5,15^ This paper aims to provide such insight for two materials in the La_1–*x*_Sr*_*x*_*Fe_1–*y*_Mn*_*y*_*O_3−δ_ family that have been used in memory reactor applications: La_0.6_Sr_0.4_FeO_3−δ_ (LSF641) and La_0.6_Sr_0.4_Mn_0.67_Fe_0.33_O_3−δ_ (LSFM). The average and local structures of these materials were studied using Bragg diffraction and total scattering (PDF)^16−19^ methods in both their oxidized (δ ≈ 0, B^3.4+^ average) and reduced (δ ≈ 0.2, B^3+^) states. Despite being derived from the relatively simple perovskite structure, the structural chemistry is complicated by the simultaneous presence of A- and B-site disorders, mixed oxidation states (B^3+^/B^4+^), which potentially lead to Jahn–Teller active d^*n*^ configurations and/or charge ordering, and oxygen vacancies. These factors lead to a number of phenomena such as metal–insulator transitions, charge ordering, charge density waves, and spin density waves in related compositions.^20−27^ As discussed below, oxygen vacancies are known to order in various ways in related systems, and their interactions influence oxygen mobility.^28^ A number of model compounds were therefore studied in parallel including LaFeO_3_, as the simplest closely related Fe^3+^ perovskite, and La_0.5_Sr_0.5_FeO_3_ (LSF551) and La_0.5_Sr_0.5_FeO_2.75_ (LSF551-red; -red = reduced here and elsewhere), which contain B^3.5+^/B^3+^, respectively, and a simpler A-site composition. Scattering data were also measured for three strontium ferrites with known local vacancy ordering patterns: Sr_8_Fe_8_O_23_ (Fe^3.75+^), Sr_4_Fe_4_O_11_ (Fe^3.5+^), and brownmillerite Sr_2_Fe_2_O_5_ (Fe^3+^), which can be expressed as SrFeO_3−δ_ with δ of 0.125, 0.25, and 0.5, respectively.

Due to its important electroceramic applications, there have been a number of previous studies on the oxygen nonstoichiometry and average structure of LSF641. Dann et al. have reported a phase diagram for La_1–*x*_Sr*_*x*_*FeO_3−δ_ for a wide range of *x* and δ, showing that various structures occur for different Sr contents.^29^ Of most relevance to the present investigation, the structures of oxidized LSF641 and LSF551 are reported as rhombohedral, while reduced LSF641 is reported as cubic. Fossdal and co-workers present a similar phase diagram showing a rhombohedral-to-cubic transition at elevated temperatures for both LSF641 and LSF551.^30^ Kuhn et al. have studied the composition and structure of LSF641 as a function of temperature and oxygen partial pressure (*p*O_2_).^31^ They also described a phase transition from rhombohedral to cubic at high temperatures and a plateau at δ = 0.2 in oxygen content as a function of *p*(O_2_), coinciding with [Sr]/2. Equivalent studies on La_0.5_Sr_0.5_FeO_3−δ_ (LSF551) at δ = 0.25 have also been reported.^32,33^ De Leeuwe et al. have reported in situ neutron diffraction measurements of LSF641 under different oxidizing and reducing buffer gases to determine cell parameters and oxygen nonstoichiometry under conditions corresponding to the cycle end points of a CL memory reactor.^13,14^

The crystal structures of the SrFeO_3−δ_ compositions with 0 ≤ δ ≤ 0.5 have been widely investigated with early studies by Greaves et al.,^34^ Takeda et al.,^35^ and Mizusaki et al.^36^ Hodges et al.^37^ reported the generally accepted structures for SrFeO_3−δ_ with δ of 0.125 (Sr_8_Fe_8_O_23_) and 0.25 (Sr_4_Fe_4_O_11_). *I*4/*mmm* SrFeO_2.875_ (Figure 1c) contains octahedral FeO_6/2_ and square-planar FeO_5/2_ polyhedra in a 3:1 ratio, with FeO_5/2_ polyhedra linked in “bow-tie” dimers. The *Cmmm* structure of SrFeO_2.75_ contains vertex-linked FeO_6_ octahedra in one-dimensional chains interconnected by similar (FeO_5/2_)_2_ bow-tie dimers (Figure 1d). The structure of SrFeO_2.5_ (Figure 1e) was identified as a brownmillerite by Gallagher et al. and contains alternating layers of edge-shared FeO_6/2_ octahedra and FeO_4/2_ tetrahedra.^38^ As with several brownmillerites, there has been some controversy concerning the true space-group symmetry, which is related to the (dis)ordering of the tetrahedral chains within and between layers.^39^ Hodges et al. obtained refinements of similar quality using both *Icmm* and *Ibm2* models and concluded that *Icmm* was more appropriate, while Schmidt and Campbell found that the structure was better described in *Ibm2* or with the Shubnikov group *Ib′m′2* when fitting neutron data.^39^ Auckett et al. have discussed these issues in detail and describe how *Ibm2* allows purely left (or right) ordered tetrahedral chains while *Icmm* has left/right disorder. They proposed a *Pbma* supercell model to better describe the long-range ordering of the tetrahedral chains.^40,41^ This was disputed by Maity et al., who stated that *Icmm* is the most appropriate model.^42^

In this paper, we describe structural studies on seven compositions in the La_1–*x*_Sr*_*x*_*Fe_1–*y*_Mn*_*y*_*O_3−δ_ family using both Rietveld refinement to probe the average structure and PDF methods to probe the local structure. Compositions are determined by diffraction methods and verified by chemical analysis. Magnetic structures are determined for each phase and ordering temperatures of LSF641 and LSFM determined by high-temperature neutron diffraction.

## 2 Experimental Section

### 2.1 Material Synthesis

Fully oxidized samples of La_1–*x*_Sr*_*x*_*Fe_1–*y*_Mn*_*y*_*O_3−δ_ were synthesized using a modified Pechini method.^43^ The following samples were prepared: LaFeO_3_ (*x* = 0, *y* = 0), LSF641 (La_0.6_Sr_0.4_FeO_3−δ_, *x* = 0.4, *y* = 0), LSF551 (La_0.5_Sr_0.5_FeO_3−δ_, *x* = 0.5, *y* = 0), and LSFM (La_0.6_Sr_0.4_Fe_0.67_Mn_0.33_O_3−δ_, *x* = 0.4, *y* = 0.33). Portions of each sample were reduced in a thermogravimetric analyzer (TGA, Rubotherm) under specific buffer gas compositions to target specific values of oxygen nonstoichiometry. Reduced LSF641 (3 – δ = 2.82 ± 0.04) and reduced LSF551 (3 – δ = 2.78 ± 0.04) were prepared by treatment at 600 °C with a buffer gas of 1:1 CO:CO_2_ (2.5 mol % each in Ar). Reduced LSFM (3 – δ = 2.79 ± 0.01) was prepared by treatment with a 1:5 CO_2_:CO buffer gas (1 mol % CO_2_, 5 mol % CO, and Ar balance) at 720 °C. These reduction conditions were chosen based on the work of Kuhn et al.,^31^ Yoo et al.,^33^ and Ungut.^44^ The final oxygen content of the reduced samples was assessed with iodometric titration based on the methods described by Murray et al.,^45^ Birkner et al.,^46^ and Tali.^47^

SrFeO_3−δ_ samples were synthesized via ball milling of strontium carbonate with iron(III) oxide followed by high-temperature calcination in air at 1200 °C. This method was described by Tofield et al.^48^ and Hodges et al.^37^ and initially yielded SrFeO_2.75 ± 0.01_. Schmidt described how the nonstoichiometry of strontium ferrites varies with high-temperature annealing and oxygen *p*(O_2_).^49^ Following this information, a sample was equilibrated in a TGA under oxygen (20 mol % in Ar) at 450 °C yielding SrFeO_2.84 ± 0.03_. Finally, a sample of SrFeO_2.48 ± 0.02_ was synthesized by reduction of SrFeO_2.75 ± 0.01_ with hydrogen (5 mol % in Ar) at 500 °C, following Hodges et al.^37^

### 2.2 Neutron Powder Diffraction Data

Samples were loaded into either 6 or 8 mm-diameter vanadium cans, and powder neutron diffraction data collected at room temperature using the POLARIS diffractometer at the ISIS Neutron and Muon Facility. Data were collected in sets of 200 μAh (typically lasting for 1 h) for Rietveld analysis. Data from different detectors were processed to produce powder patterns labeled as banks 1–5 according to their mean 2-theta angle, *d* spacing ranges, and δ*d*/*d* resolution (banks 1, 2, 3, 4, and 5 centered at 10.44, 25.99, 51.99, 91.51, and 145.94° 2-theta, respectively). Crystallographic models were Rietveld-refined against the data from these banks using TOPAS Academic version 7.^50−52^ Unit-cell parameters, oxygen site occupancy, atomic displacement parameters (including anisotropic for oxygen), and sample contributions to the peak shape describing isotropic size and strain were refined where appropriate. Experimental background was fitted using a Chebyshev polynomial. The instrumental contribution to the peak shape was determined empirically from a Si standard. The B-site magnetic moment was refined for samples that exhibited magnetic ordering by using the appropriate Shubnikov magnetic space group for G-type ordering of the nuclear structure. For some samples, a second Pawley-fitted phase was introduced to fit minor Bragg peaks originating from the vanadium sample can. The cubic *Im*3̅*m* model reported by Karen and co-workers (ICSD Collection Code 171003) was used.^53^ Variable-temperature data were recorded for samples of LSF641 and LSFM held in a steel can in a RAL furnace. Heating data were collected on oxidized LSF641 and LSFM. Cooling data were collected on reduced LSF641-red and LSFM-red prepared by reduction under extended chemical looping conditions of H_2_O/CO feeds for LSF641 and CO_2_/CO for LSFM; each feed gas was 5% in Ar.

### 2.3 Pair Distribution Function Analysis

High-quality data sets were also collected at room temperature for pair distribution function (PDF) analysis. These were collected in eight sets of 200 μAh (approximately 8 h per each sample). The background scattering due to the empty furnace and the empty can was measured at room temperature for 1–2 h each. Data were summed, normalized onto an absolute scale, and processed using GudrunN software.^54^ The *D*(*r*) functions (.mdor01 file format) were produced using a *Q*_max_ of 36 Å^–1^.

Synchrotron PXRD data were collected at the high-resolution powder diffraction beamline ID22 at the European Synchrotron Radiation Facility, ESRF. Samples were loaded into 1 mm borosilicate capillaries and spun during collection using λ = 0.354282 Å X-rays. Data were collected by scanning the multianalyzer detector from −10 to 132° at a speed of 6° min^–1^. Two further high-angle collections were performed from 20–132° and 40–132° to give better statistics at high *Q*. The total data collection time was approximately 1.5 h per sample. Data were binned to a 0.0002° resolution and PDFs produced using PDFGetX3 for *Q* = 0–28 Å^–1^ with *r*_step_ = 0.01 and *r*_poly_ = 0.8.^55^

Both small-box and big-box local structure refinements were performed using TOPAS Academic version 7.^50−52^ Small-box refinement used the same crystallographic models used for fitting Bragg data. Mixed occupancy sites were approximated using a single element with the weighted average scattering length of the two elements. *r*-dependent isotropic atomic displacement parameters were used to account for the correlated atomic motion of directly bonded atoms giving sharper PDF peaks at low *r*.^17^

Big-box models were constructed by expanding unit cells into supercells containing several thousand atoms. Where necessary, oxygen vacancies were randomly generated then swapped using a bespoke Monte Carlo routine to target specific ratios of B-site coordination geometries. Models were created targeting mixtures of octahedral (BO_6_) and square-pyramidal sites (BO_5_) or octahedral and tetrahedral (BO_4_) sites. The atomic coordinates in the supercells were refined against neutron-only or neutron and X-ray PDF data over a 0.2–21.0 Å range and simultaneously against bond valence sum (BVS) and bond angle restraints to keep local geometries chemically sensible. The average shifts of B-site coordinates were restrained to prevent the entire structure translating in the *P*1 unit cell. Weights on the experimental PDF were set to 0 below 1.65 Å to avoid fitting artifacts from the Fourier transform. A low weighting was set on chemical restraints to ensure that the fit was driven predominantly by the experimental data. Neutron Bragg data were fitted simultaneously by folding supercell coordinates into a small subcell compatible with the average structure and assigning appropriate partial site occupancies. Bragg data from POLARIS bank 4, which offers a good balance of resolution and coverage of reciprocal space, were fitted over a ToF range of 2800–20,000 μs (*d* spacing 0.5–3.7 Å). In the initial stages of refinement, coordinate changes were limited to prevent unreasonably large atomic shifts. These constraints were removed during later stages. For magnetically ordered samples, the magnetic contribution to the Bragg data was included in the fit based on values derived in standard Rietveld refinement. Refinements typically converged within 1–2 h on a standard desktop PC and were then subjected to randomization and rerefinement to ensure convergence.

## 3 Results and Discussion

### 3.1 Average Structure

**Figure 2:**
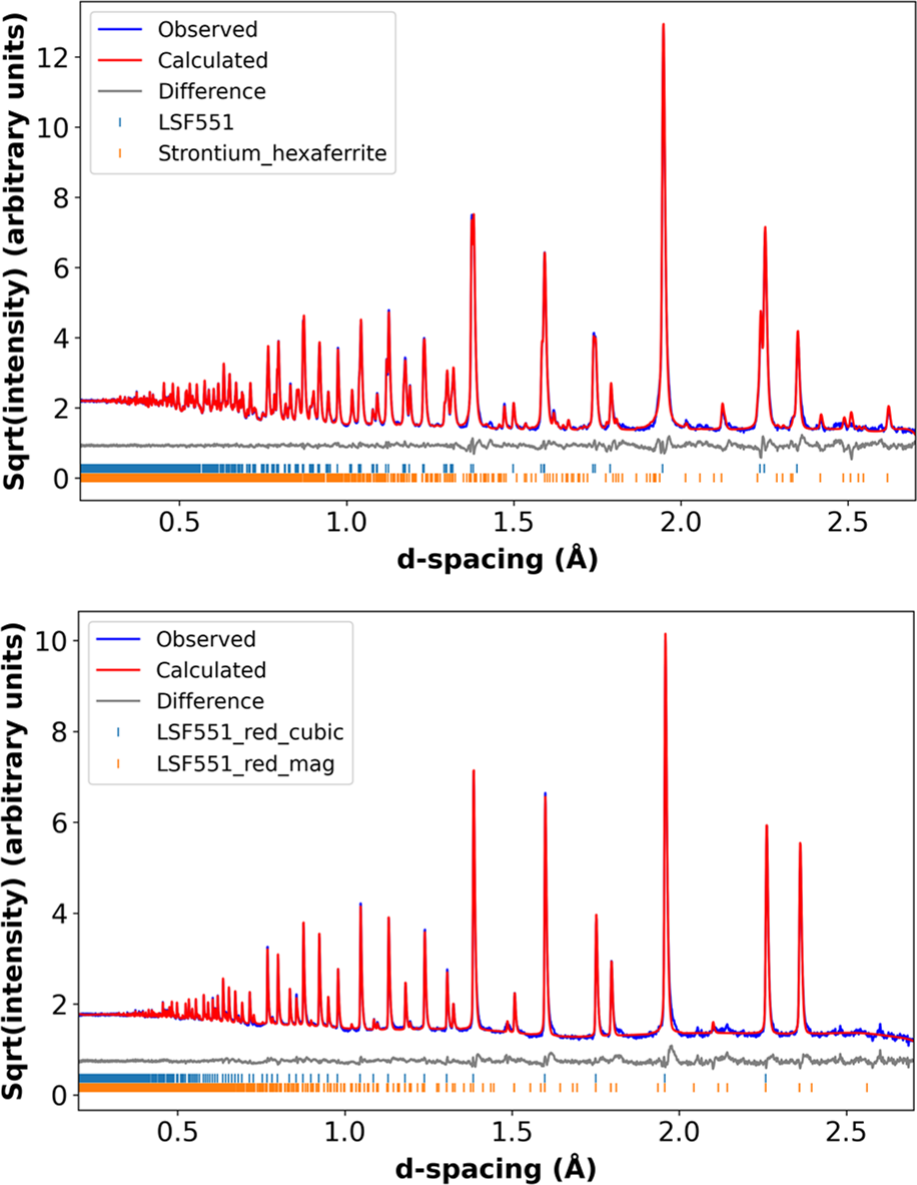
Rietveld fits of bank 5 data for LSF551 (top, *R*3̅*c*) and reduced LSF551 (bottom, *Pm*3̅*m*). Blue, red, and gray lines show observed, calculated, and difference plots, respectively. Data shown on a  scale to emphasize weaker features.

Rietveld refinements to determine the nuclear structure, magnetic structure, and oxygen content of each sample were performed using all five banks of neutron data from the POLARIS diffractometer. An example Rietveld fit is given in Figure 2, and full CIF files are provided in the Supporting Information. Metal site occupancies were refined where appropriate and revealed A- and B-site ratios consistent with the target values so were typically fixed for final refinements. For oxidized LSF551, a secondary phase of strontium hexaferrite (SrFe_12_O_19_) was present at approximately 5 wt % and was modeled using the structure reported by Kimura et al. (ICSD 69023).^56^ For the other oxidized samples (LaFeO_3_, LSF641, and LSFM), the results showed that essentially phase-pure materials had been synthesized, and average crystal structures were consistent with those reported by Dann and co-workers.^29^ For LSF641, evidence of partial segregation into two closely related phases could be seen in the highest resolution bank, consistent with a previous report.^14^

Magnetically ordered samples all showed G-type antiferromagnetic ordering (all Fe nearest neighbor interactions antiferromagnetic). LaFeO_3_ (Shubnikov group *Pn′ma′*) gave a refined magnetic moment of 4 μ_B_ comparable to literature values reported by Yang et al.^57^ and Götsch et al.^58^ and consistent with the expected value for high-spin Fe^3+^ with covalency effects taken into account. The other fully oxidized samples were described in Shubnikov group 167.103 (unified symbol^59^*R*3̅*c*.1[*R*3̅*m*]) with the moment along the *c*-axis. Refined moments were significantly lower, with values for LSF641 similar to those previously reported.^57^ This is consistent with their increased B^4+^ content, which would both decrease expected saturated moments and disrupt strong Fe^3+^–O–Fe^3+^ d^5^–d^5^ antiferromagnetic superexchange. We note, for example, that SrFeO_2.75_ (Fe^3.5+^) orders magnetically below room temperature, on only one of the two Fe substructures.^60^ Rapid variable-temperature neutron powder data recorded on heating under Ar (conditions where minimal oxygen loss occurs) gave Néel temperatures of 350 and 275 °C for LSF641 and LSFM, respectively (see the Supporting Information).

Rietveld fitting for reduced samples LSF641-red, LSF551-red, and LSFM-red gave similarly good agreement using either cubic (*Pm*3̅*m*) or rhombohedral (*R*3̅*c*) models. Complementary synchrotron XRD data revealed no significant intensity for rhombohedral ordering peaks, and the cubic model was therefore used. Refined ABO_3−δ_ compositions were consistent with those obtained by iodometric titration analysis, yielding La_0.6_Sr_0.4_FeO_2.834(5)_, La_0.5_Sr_0.5_FeO_2.740(6)_, and La_0.6_Sr_0.4_Fe_0.67_Mn_0.33_O_2.84(1)_. Magnetic moments of LSF641-red (3.99 μ_B_) and LSF551-red (3.86 μ_B_) were comparable to LaFeO_3_ (3.99 μ_B_), consistent with full reduction to Fe^3+^. LSFM-red showed a lower moment (2.46 μ_B_), consistent with d^4^ Mn^3+^ dilution of the B-site. Néel temperatures of B^3+^-containing LSF641-red and LSFM-red collected on cooling from high temperatures were higher than the oxidized samples as expected at ∼500 and ∼425 °C, respectively. This is consistent with the higher Fe^3+^ content.

The Rietveld refinements for SrFeO_2.875_ (Sr_8_Fe_8_O_23_), SrFeO_2.75_ (Sr_4_Fe_4_O_11_), and SrFeO_2.5_ (Sr_2_Fe_2_O_5_) reference samples showed good agreement with previously reported models.^25,37^ For the first two samples, clear peaks due to vacancy ordering were observed, though their width relative to other reflections suggested domain size broadening. Refinements of SrFe_2_O_5_ suggested an approximately 50:50 mixture of left- and right-oriented FeO_4_ tetrahedra, implying layer-to-layer disorder as discussed in the Introduction. The magnetic structure of SrFe_2_O_5_ was consistent with previous reports, with moments on each site (3.75(3) and 3.67(3) μ_B_) close to values expected for Fe^3+^.

### 3.2 Local Structure: Experimental Observations and Small-Box Modeling

**Figure 3:**
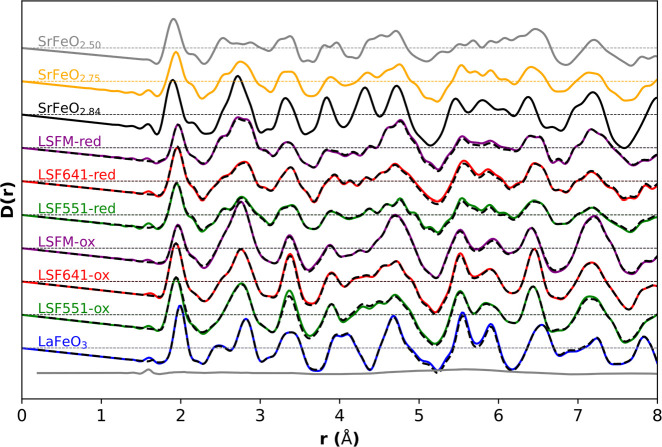
Solid lines show 300 K neutron PDF data for all samples. Oxidized/reduced pairs with the same metal composition are shown with the same color lines. Dashed black lines superimpose big-box fits for selected data sets.

The experimental neutron PDF data for all 10 samples are compared in Figure 3. The data are on a consistently prepared, well-characterized set of related compositions, measured and processed using an equivalent protocol. As such, they give an opportunity to compare the changes in local structure across related compounds with systematically changing compositions, oxygen contents, and B-site oxidation states.

As anticipated from the Rietveld refinements, LaFeO_3_ shows the simplest PDF with a sharp Fe–O peak around 1.99 Å, followed by sharp features due to O–O and La–O distances. Introduction of La_0.6_Sr_0.4_ and La_0.5_Sr_0.5_ A-site disorder in LSF641 and LSF551 leads to significant broadening of peaks in the 2.3–3 and 3.5–5.2 Å regions, with the two compositions showing similar PDFs as expected. Comparing LSF641 and LSFM, which differ only in the B-site composition changing from Fe_1.0_ to Fe_0.67_Mn_0.33_, we see significant changes in the PDF due to the negative scattering length of Mn (*b*_Fe_ = 9.45 fm, *b*_Mn_ = −3.73 fm). This helps highlight local chemical changes as discussed later.

The corresponding reduced samples La_0.6_Sr_0.4_FeO_2.8_, La_0.5_Sr_0.5_FeO_2.75_, and La_0.6_Sr_0.4_Fe_0.67_Mn_0.33_O_2.8_ all show broadening of the Fe–O peak and growth of a high-*r* shoulder at around 2.1 Å. Similar features are seen in the first peak of the PDFs of SrFeO_2.875_, SrFeO_2.75_, and SrFeO_2.5_, which are known to contain mixed iron polyhedra of 3:1 Oh:SqPy (SqPy = square pyramid), 1:1 Oh:SqPy, and 1:1 Oh:Td, respectively. The Fe-only PDFs show a strong similarity to SrFeO_2.75_ over the whole *r* range, suggesting similar local structures.

**Figure 4:**
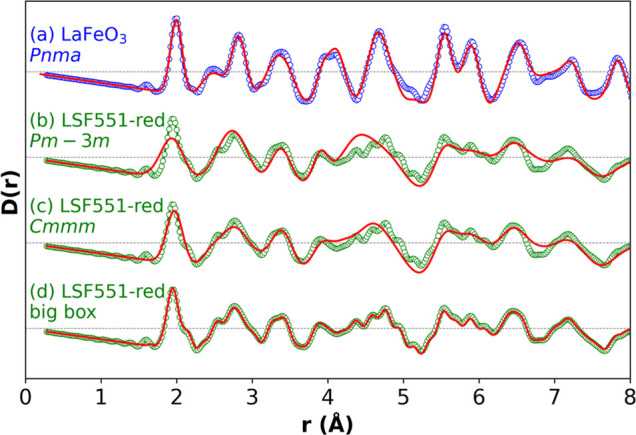
Small-box PDF fitting using models discussed in the text for (a) LaFeO_3_ and (b, c) La_0.5_Sr_0.5_FeO_2.75_ (LSF551-red). Small-box fits in (b) and (c) do not reproduce the observed experimental features. (d) Big-box fit for La_0.5_Sr_0.5_FeO_2.75_.

Small-box fits to selected data using conventional crystallographic models are included in Figure 4. For the simplest LaFeO_3_ composition, good agreement is observed between the experimental data and the PDF calculated from the average Rietveld structure with isotropic displacement parameters (Figure 4a). Note that any magnetic contributions to the PDF are not included in this fit. The rapid fall-off in magnetic scatter with *Q* due to the form factor means that magnetic contributions to the PDF are generally weak and appear as a broad oscillating contribution to the background.

For substituted samples and those with oxygen vacancies, small-box fitting is significantly worse. For example, Figure 4b shows a fit of the average cubic Rietveld model to PDF data of La_0.5_Sr_0.5_FeO_2.75_. The average structure (which gives an excellent fit to the Bragg data) gives a poor fit to the PDF and fails to reproduce both short- and long-range features. Models related to the *Cmmm* and *Ibm*2 structures of SrFeO_2.75_ and SrFeO_2.5_, which contain square-pyramidal and tetrahedral Fe sites, respectively, were also tested. While giving a better fit (e.g., Figure 4c), they fail to reproduce all features of the experimental data.

### 3.3 Big-Box Modeling Methods

As discussed above, small-box modeling is incapable of describing all the features in the PDF of vacancy-containing or substituted materials. We have therefore used big-box fitting to probe local structures. In this approach, we use a supercell containing between 512 and 729 perovskite subcells (2560–3500 atoms) to fit the PDF *D*(*r*) data, fold the supercell onto an appropriate subcell to fit the neutron Bragg data from POLARIS bank 4, and use soft bond valence and angle restraints to retain a chemically sensible model. Bond valence restraints were chosen in preference to bond length restraints as they allow chemically sensible polyhedral distortions to occur. For selected compositions, we simultaneously fitted X-ray PDF data, and for magnetically ordered samples (Table 1), the magnetic contribution to the Bragg data was included based on the Rietveld refinements described in Section 3.1. Isotropic atomic displacement parameters for all atoms were set to the artificially low value of 0.01 Å^2^ so that peak broadening in the PDF and intensity fall-off with *Q* for the Bragg data were modeled through the distribution of coordinates in the superstructure.

**Table 1:** 295 K Rietveld Refinement Results and Oxygen Composition by Iodometric Titration

sample ID and target composition	space group^a^	*R*_wp_ (%), GOF	lattice parameters (Å)	*a*_p_ (Å)^d^	composition by Rietveld refinement^b^	magnetic moment (μ_B_), *T*_N_	oxygen content by titration
1. LaFeO_3_	62.448	2.23, 2.18	*a* = 5.56115(7), *b* = 7.85484(11), *c* = 5.55765(8)	3.9299	LaFeO_2.984(1)_	3.99(1)	2.99 ± 0.01
2. LSF641 La_0.6_Sr_0.4_FeO_3_	167.103	2.76, 3.27	*a* = *b* = 5.52144(4), *c* = 13.42429(10)	3.8946	La_0.61(1)_Sr_0.39(1)_FeO_3.007(3)_	1.17(1), *T*_N_ = 350 °C	3.02 ± 0.04
3. LSF551 La_0.5_Sr_0.5_FeO_3_	*R*3̅*c*	2.88, 3.36	*a* = b=5.51408(3), *c* = 13.41775(11)	3.8905	La_0.5_Sr_0.5_FeO_3.008(2)_	N/A	2.94 ± 0.02
4. LSFM La_0.6_Sr_0.4_Fe_0.67_Mn_0.33_O_3_	167.103	3.09, 3.89	*a* = *b* = 5.50831(5), *c* = 13.4023(2)	3.8662	La_0.6_Sr_0.4_Fe_0.67_Mn_0.33_O_2.998(3)_	1.091(8), *T*_N_ = 275 °C	2.94 ± 0.01
5. red. LSF641 La_0.6_Sr_0.4_FeO_2.8_	*Pm*3̅*m*/167.108	3.96, 3.64	*a* = 3.914623(12)	3.9146	La_0.6_Sr_0.4_FeO_2.834(5)_	3.989(7), *T*_N_ = 500 °C	2.82 ± 0.04
6. red. LSF551 La_0.5_Sr_0.5_FeO_2.75_	*Pm*3̅*m*/167.108	3.69, 3.45	*a* = 3.9129(1)	3.9129	La_0.5_Sr_0.5_FeO_2.740(4)_	3.855(8)	2.78 ± 0.04
7. red. LSFM La_0.6_Sr_0.4_Fe_0.67_Mn_0.33_O_2.8_	*Pm*3̅*m*/167.108	3.74, 3.28	*a* = 3.9069(4)	3.9069	La_0.6_Sr_0.4_Fe_0.67_Mn_0.33_O_2.84(1)_	2.46(1), *T*_N_ = 425 °C	2.79 ± 0.01
8. SrFeO_2.875_	*I*4/*mmm*	2.75, 2.85	*a* = *b* = 10.92844(8), *c* = 7.71528(12)	3.8617	SrFeO_2.875_	N/A	2.84 ± 0.03
9. SrFeO_2.75_	*Cmmm*	3.31, 3.92	*a* = 10.9419(4), *b* = 7.71936(9), *c* = 5.47001(19)	3.8653	SrFeO_2.75_	N/A	2.75 ± 0.01
10. SrFeO_2.5_	*Ima2*^c^/30.122	3.26, 2.96	*a* = 15.6185(2), *b* = 5.66627(8), *c* = 5.52825(9)	3.9210	SrFeO_2.5_	Fe1 3.75(3), Fe2 3.67(3)	2.48 ± 0.02

a Shubnikov groups used for magnetic structures; / indicates Shubnikov group of separate magnetic phase; the standard setting of space group 62 is *Pnma*; space group 167 is *R*3̅*c*.

b Where no estimated standard deviation is shown, the occupancy was fixed at the expected value.

c Nonstandard setting of the *Ibm2* space group discussed in the text, partial occupancy of left- and right-ordered tetrahedral chains.

d *a*_p_ is (volume*)*^1/3^ normalized to a single perovskite cubic cell.

For each compound, fits were started with coordinates randomly displaced from an ideal perovskite geometry and least-squares refined to convergence. Several cycles of randomization of coordinates around their converged values and rerefinement were then performed to find the best-fit model. Despite the complexity and size of the models, convergence could typically be achieved in a few hours on a standard desktop PC, and refinements started from different random starting points converged to equivalent models. This approach is similar to that used in software such as RMCProfile^61^ to produce models consistent with both the local and long-range structures but uses direct least-squares minimization rather than reverse Monte Carlo modeling.

**Figure 5:**
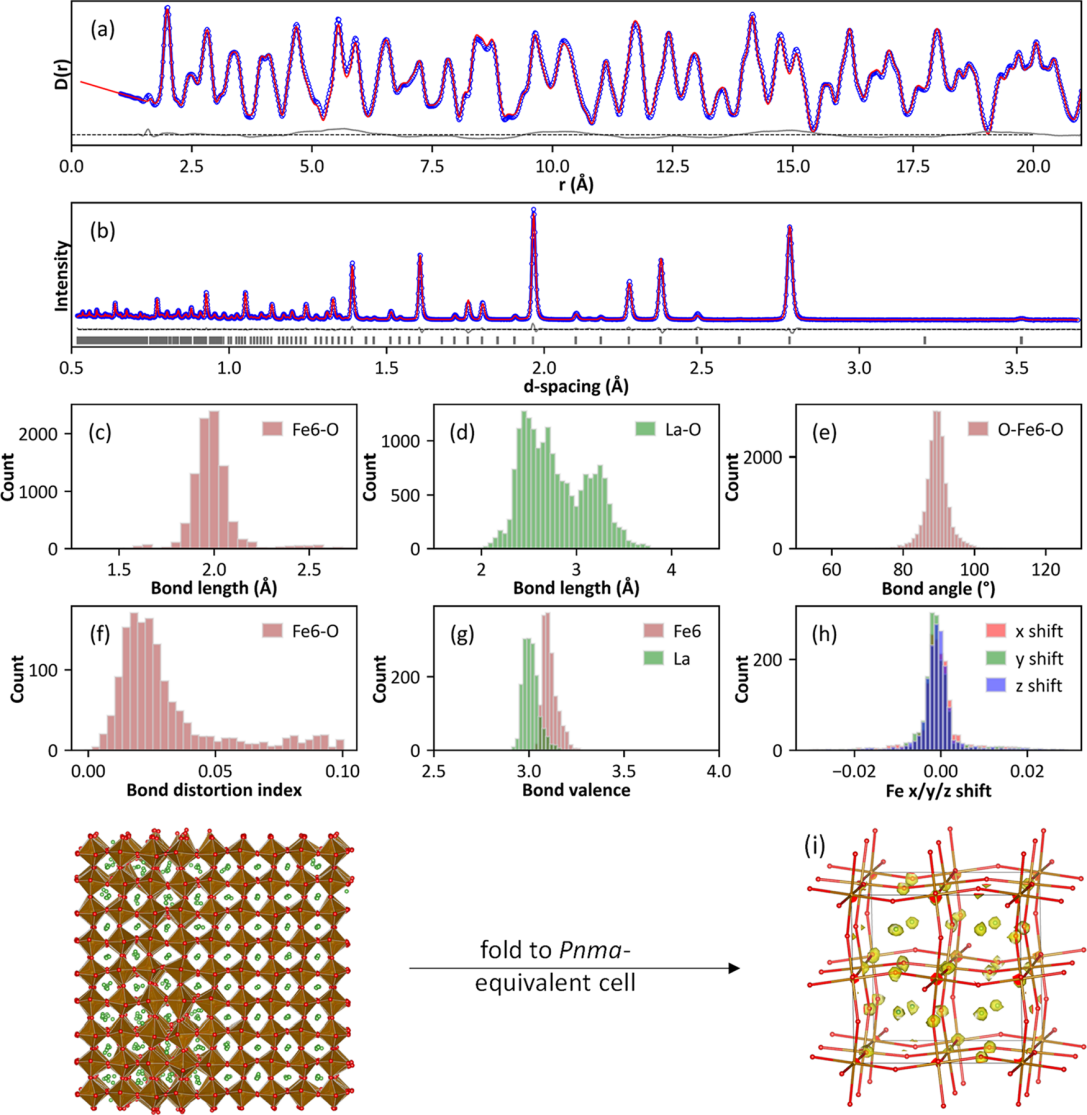
Simultaneous fits to neutron (a) PDF and (b) Bragg data (bank 4); observed, calculated, and difference plots in blue, red, and gray, respectively. In (b), gray vertical bars show predicted reflection positions. (c–g) Histograms of parameters extracted from the model: (c) Fe–O bond lengths, (d) La–O bond lengths, (e) O–Fe–O 90° bond angles, (f) bond distortion index, and (g) Fe and La bond valence sums. Panel (h) shows the range of Fe shifts in fractional coordinates; the sum was restrained to zero to prevent origin shifts. Panel (i) shows a single supercell configuration (one of many consistent with the data) and the cloud (yellow) of positions that results from folding three configurations from different refinements onto a 2 × 2 × 2 unit cell; the Rietveld model is superimposed.

Figure 5 includes a summary of results for the simplest system studied, vacancy-free LaFeO_3_, to demonstrate the method. This fit used a close-to-metrically cubic 8 × 8 × 8 superstructure of the basic perovskite cell containing 2560 atoms. Excellent fits to the PDF (Figure 5a) and Bragg (Figure 5b) data were obtained, with the former showing only a smoothly oscillating difference curve, which includes the small unmodeled magnetic contribution to the PDF. Histograms in Figure 5c–f show bond distance and angle distributions, the bond distortion index for all 1536 FeO_6_ octahedra, and bond valence sums for Fe and La; these were averaged from three different starting configurations. The bond distortion index plotted is defined as  and gives an indication of the degree of local distortion.^62^ Bond valence sum histograms with means/standard deviations of 3.07/0.11 and 2.95/0.18 for Fe and La, respectively, are fully consistent with values of 3.08 and 2.95 from the Rietveld average model. The Fe–O–Fe bond angle distribution (shown later in Figure 7d), which is a measure of polyhedral tilting, has an average of 156.2° (standard deviation 5.4°) compared to discrete angles of 155.7 and 157.3° in the Rietveld model. Figure 5i shows the “cloud” of atomic positions obtained by folding the superstructure models back onto a 2 × 2 × 2 perovskite unit cell superimposed on the *Pnma* structure obtained by Rietveld refinement in an equivalent setting. The yellow clouds encompass all grid voxels (each 0.2 Å^3^) containing ≥2 atoms (a perfect undistorted superstructure would have 168 atoms in a single voxel). The big-box model successfully extracts the average atomic positions, the coupled polyhedral tilting, and the range of local distortions normally captured by atomic displacement parameters.

Similar fits were performed for all samples. For the three oxidized samples (LSF641, LSF551, and LSFM), a 6 × 6 × 3 supercell of the crystallographic cell with *a* = *b* ≈ 33.1 Å, *c* ≈ 40.2 Å, α = β = 90°, and γ = 120° containing 3240 atoms was used. La sites were randomly swapped to Sr to give compositions of La_389_Sr_259_ and La_324_Sr_324_ for La_0.6_Sr_0.4_ and La_0.5_Sr_0.5_, respectively. The three corresponding reduced samples were modeled using a 9 × 9 × 9 supercell of the basic cubic perovskite cell, which contains up to 3645 atoms. A-site occupancies were set using the same protocols as for the oxidized samples. Oxygen vacancies were introduced using a bespoke Monte Carlo routine. Starting with an ideal perovskite composition, the appropriate number of oxygen vacancies was introduced at random (145 for ABO_2.8_ and 182 for ABO_2.75_). Vacancies were then swapped pairwise to produce configurations that maximized either the number of BO_6_ octahedra and BO_5_ square pyramids (mimicking the structural motifs in SrFeO_2.875_ and SrFeO_2.75_) or the number of BO_6_ octahedra and BO_4_ tetrahedra (mimicking SrFeO_2.5_). The connectivity of the BO*_n_* polyhedra and the initial random allocation of vacancies means that the target polyhedral pattern cannot always be achieved perfectly. However, in the models discussed, maximizing square pyramids for La_0.5_Sr_0.5_FeO_2.75_ gave 367 octahedra, 361 square pyramids, and a single three-coordinate B-site. Maximizing tetrahedra gave six-:five-:four-coordinate Fe in a 546:2:181 ratio; of the 181 four-coordinate sites, 165 have cis vacancies, which can lead to tetrahedral coordination and 16 trans vacancies, which lead to square-planar. For La_0.6_Sr_0.4_Fe_0.67_Mn_0.33_O_2.8_, square-pyramidal vacancy models were generated in which the Mn were predominantly MnO_6_ (i.e., vacancies associated with Fe), MnO_5_ (vacancies associated with Mn), or a statistical mixture of both.

Example fits for La_0.5_Sr_0.5_FeO_2.75_ based on a combination of FeO_6_ octahedra and FeO_5_ square pyramids to X-ray and neutron PDF data and neutron Bragg data are shown later in Figure 8, and others are in the SI. Extracted parameters from all models are discussed in the following sections. In Section 3.4, we discuss the oxidized samples, and in Section 3.5 the reduced.

### 3.4 Discussion: Local Structure of Oxidized Samples

**Figure 6:**
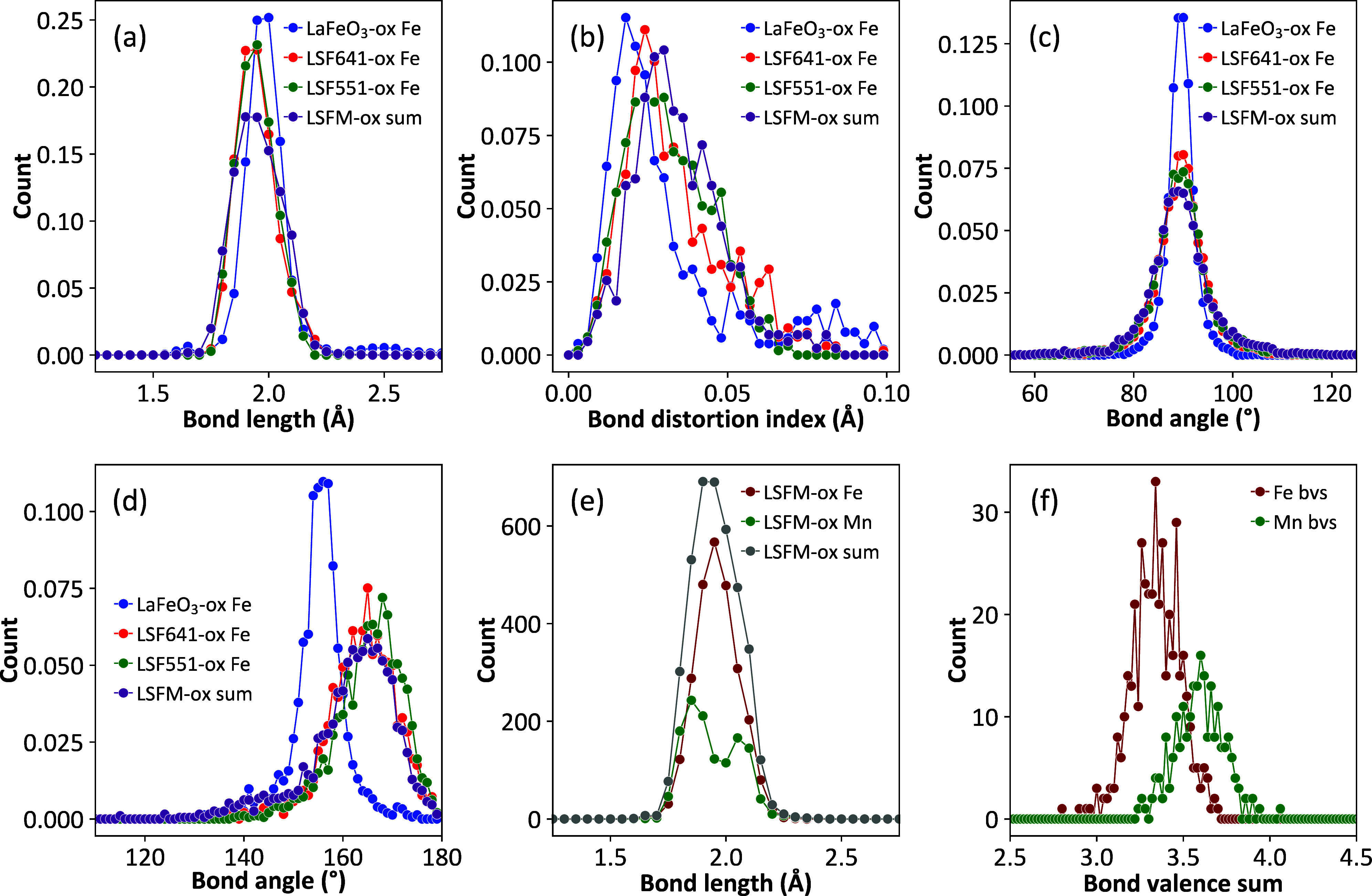
Comparison histograms for big-box PDF fitting of oxidized samples. (a) B–O distance, (b) bond distortion index, (c) O–B–O angle, (d) B–O–B bond angles, (e) B–O distance for LSFM, and (f) B-site bond valence sums for LSFM. The histograms in (a)–(d) are normalized to reflect the slightly different number of B-sites in the different models.

**Figure 7:**
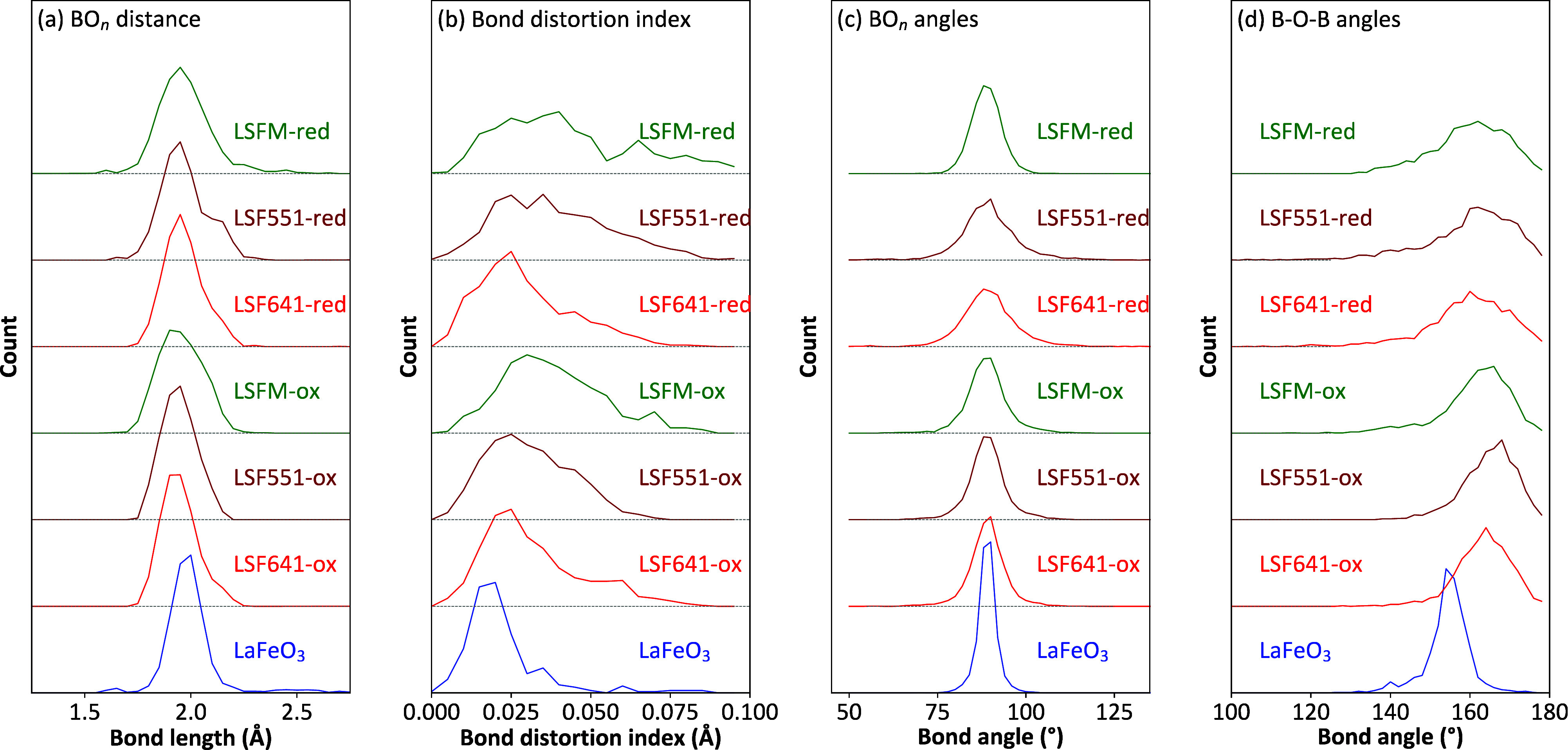
(a–d) Normalized histograms of parameters related to BO*_n_* polyhedra for all compounds.

Histograms of B–O distances, BO_6_ polyhedral bond distortion index, O–B–O angles, and B–O–B angles for the four oxidized samples (LaFeO_3_, LSF641, LSF551, and LSFM) are superimposed in Figure 6a–d (and shown offset in Figure 7). B–O bond lengths show the expected shifts to shorter distances between Fe^3+^-containing LaFeO_3_ and the Fe^3+^/Fe^4+^-containing Sr-substituted samples. There is also an increase in the octahedral distortions as evidenced by the increasing width of the bond length distribution, the increasing spread of O–B–O bond angles, and the bond distortion index (BDI) histograms. For reference, BDI values would be 0 for a perfect octahedron or around 0.04 for the strained Fe^3+^O_6_ octahedron in Sr_2_Fe_2_O_5_ or a Jahn–Teller d^4^ Mn^3+^ in LaMnO_3_. The increased distortion might be expected to arise from the presence of Jahn–Teller active d^4^ Fe^4+^, though observed distortions are low in Fe^4+^ phases such as CaFeO_3_^26,63^ and Sr_2_FeO_4_.^64^ We checked our configurations for evidence of correlated local distortions using the VanVleckCalculator described by Nagle-Cocco and Dutton^65^ but found no evidence for these. Figure 6d shows the increase in average B–O–B angles in the Sr-substituted samples. This occurs despite their average cell volumes being smaller than LaFeO_3_ and reflects significantly lower average polyhedral tilts.

Figure 6e,f separates out the FeO_6_ and MnO_6_ bond distances and bond valence sums for LSFM. Bond valence histograms show a systematically higher value for Mn (3.65) than Fe (3.45) despite both sites being restrained to the average expected oxidation state of 3.4 in the modeling. This difference is also reflected in the split peak of the Mn–O bond distance histogram, which shows a high proportion of short Mn–O distances. This double peak behavior suggests that a high proportion of Mn^4+^ is present, though it could also indicate some Jahn–Teller distorted Mn^3+^. Expected Mn–O distances are around 1.903 Å for Mn^4+^ and 2.106 Å for Mn^3+^ based on bond valence sums, with Jahn–Teller distortions leading to values between ∼1.9 and 2.2 Å for Mn^3+^. We note that the presence of Mn^4+^ has been inferred from Mössbauer data for the similar composition La_0.5_Sr_0.5_Fe_0.5_Mn_0.5_O_3_^66^ and via XANES data in LSFM^44^ and the closely related Sr_2_FeMnO_5.5_. PDF modeling of the latter using reverse Monte Carlo methods suggested that Fe and Mn were randomly distributed with predominantly five-coordinate Fe and six-coordinate Mn, as found in LSFM.^67,68^

The presence of shorter Mn–O bonds can also be inferred from the raw PDF data of Figure 3. The experimental B–O peak is both weaker and sharper in LSFM than in LSF551 and LSF641 despite their compositional similarities. Both effects are caused by the negative scattering length of Mn. This reduces the overall peak area (Mn–O contributes “negative peaks”), and the shorter Mn^4+^–O distances effectively removes the leading edge of the distribution giving a sharper experimental peak. The presence of short Mn^4+^–O distances also leads to the broadest modeled B–O distribution of the oxidized samples (Figure 7a), despite the sharp experimental peak.

### 3.5 Discussion: Local Structure of Reduced Samples

**Figure 8:**
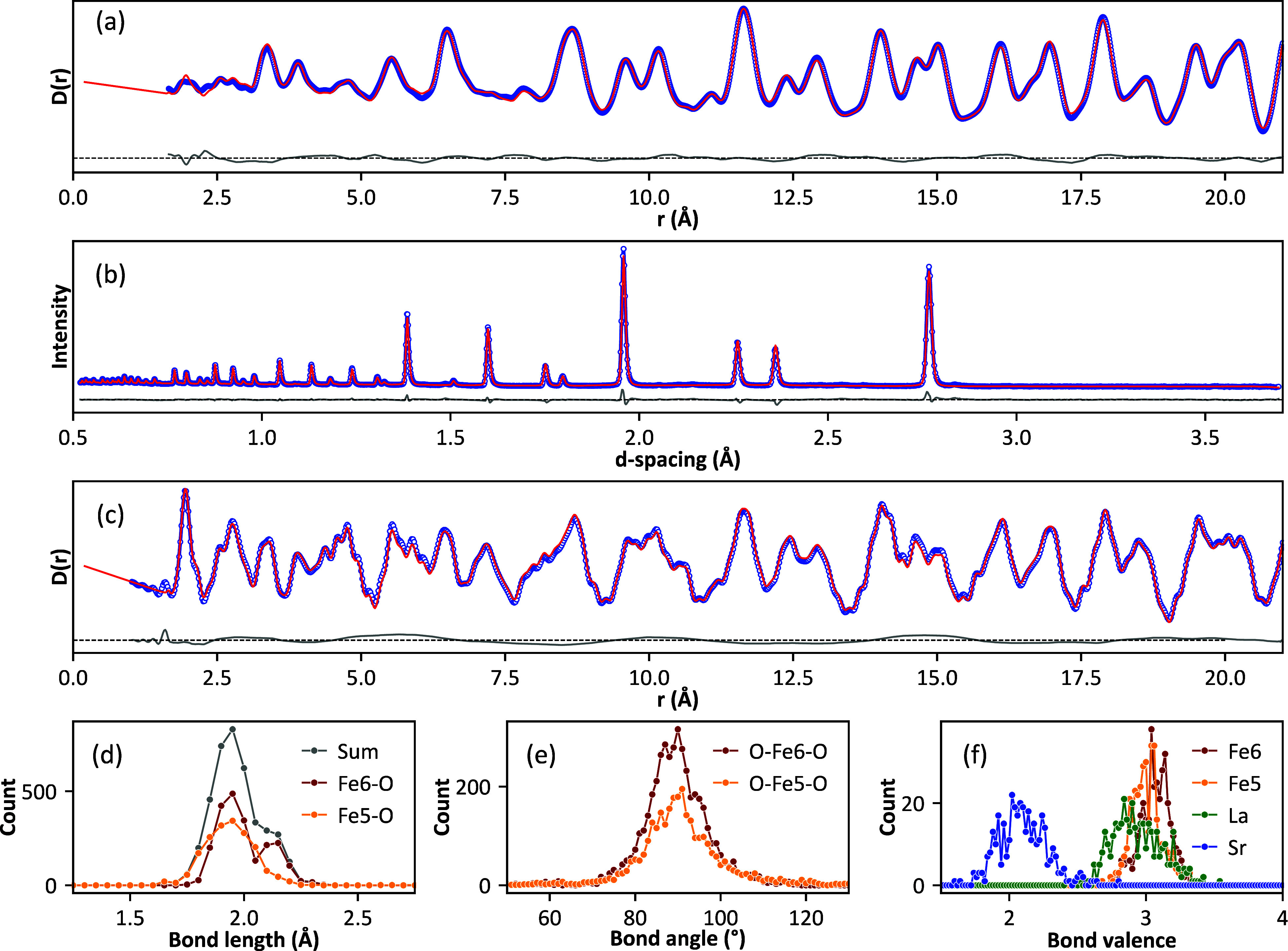
Fits to (a) X-ray PDF, (b) neutron Bragg (bank 4), and (c) neutron PDF data of LSF551-red. Lower plots show histograms of (d) FeO_5_ and FeO_6_ bond lengths, (e) O–Fe–O bond angles, and (f) bond valence sums for all cations.

Figure 8 shows fits and key parameters for La_0.5_Sr_0.5_FeO_2.75_ (LSF551-red) using a model containing a 50:50 mixture of octahedral and square-pyramidal sites. Similar plots for LSF641-red and LSFM-red are included in the SI, and the most important extracted parameters are included in Figure 7. The experimental B–O distribution (Figure 8d) is well-explained by a combination of relatively regular BO_5_ square pyramids and more distorted FeO_6_ octahedra. The distances observed are consistent with average bond lengths of ∼1.97 Å found for Fe^3+^O_5_ square-pyramidal sites in charge-ordered YBaFe_2_O_5_ and NdBaFe_2_O_5_^24^ and significantly longer than those for Fe^4+^O_5_ (around 1.87 Å) observed in SrFeO_2.875_ and SrFeO_2.75_. The observed octahedral distortion is driven by the data and is not an artifact of the restraints used. This was confirmed by analysis of models refined solely against the restraints, which showed no such splitting. As can be seen from Figure 7a, the high *d* spacing ∼2.1 Å B–O shoulder is significantly more pronounced in LSF551-red than LSF641-red. This occurs because even the relatively small composition change from ABO_2.75_ to ABO_2.8_ leads to significant changes in the ratio of octahedral to square-pyramidal sites from 1:1 to 1.0:0.66; the number of FeO_6_ octahedra adjacent to a square-pyramidal FeO_5_ is therefore significantly higher in LSF551.

We also tested a model containing 75% octahedral, 23% tetrahedral, and 2% square-planar Fe for LSF551. Accommodating local tetrahedral defects is harder than square-pyramidal as significant local distortions are required to accommodate both 90 and 109.5° O–Fe–O angles. Geometrically optimized models lead to 90° bond angle distributions with a full width at half-maximum of around 5° and tetrahedral distributions around 10°. The frustrations of polyhedral linking are somewhat alleviated in the vacancy-ordered layered brownmillerite structure of Sr_2_Fe_2_O_5_, but even there, significant polyhedral distortions occur (O–Fe–O tetrahedral angles range from 99.5 to 141.9°; Fe–O octahedral distances from 1.94 to 2.22 Å).^40^ Good fits to the experimental data could be achieved (see the SI), though the local polyhedral distortions are significantly higher than for square-pyramidal defects. We also note that the PDF of LSF551-red is visually very similar to that of square-pyramid-containing SrFeO_2.75_ (Figure 3) in the 0–8 Å range, whereas SrFe_2_O_5_ differs significantly. This suggests that square-pyramidal local defects dominate.

Models containing square-pyramidal defects also gave excellent fits to data from LSFM-red. Models were tested in which vacancies were located exclusively around Fe, predominantly around Mn (the composition means 100% Mn and 9% Fe have to be BO_5_ groups) or randomly distributed. Differences between these models were statistically insignificant, though all suggest that Mn polyhedra are more distorted than Fe. This is consistent with the presence of Jahn–Teller active d^4^ Mn^3+^. In contrast to the fitting of oxidized LSFM, bond valence sum histograms for Fe and Mn sites (see the SI, Figure S2) showed no significant difference between the metals. This is consistent with the presence of Fe^3+^/Mn^3+^ in LSFM-red and Fe^3+^/Mn^4+^ in LSFM. The observation of consistent bond valence sums in LSFM-red but not in LSFM-ox is evidence that the predominance of Mn^4+^ in LSFM-ox is real.

## 4 Conclusions

Rietveld and PDF studies have been conducted to better understand the local structure of oxidized and reduced La_1–*x*_Sr*_*x*_*Fe_1–*y*_Mn*_*y*_*O_3−δ_ compositions, which are important oxygen carrier materials for chemical looping applications. Rietveld analysis of Bragg diffraction data shows that the average structure of the oxidized materials is rhombohedral and that the reduced vacancy-containing materials are cubic. We observe excellent agreement between the Rietveld-refined oxygen content and that determined by titration methods. This means that neutron powder diffraction can be used to obtain accurate oxygen content on these materials in *operando* studies. Antiferromagnetic structures and magnetic moments for all samples are consistent with the transition metals present and their oxidation states. For the oxidized samples, we see a systematic decrease in magnetic moment with nominal Fe^4+^ content, and we see a lower moment for Mn-containing samples. Variable-temperature neutron diffraction experiments show that Néel temperatures are higher for the reduced samples than oxidized, again consistent with their Fe^3+^ content (350 °C/500 °C for LSF641-ox/-red and 275 °C/425 °C for LSFM-ox/-red).

Although Rietveld fitting shows excellent agreement between average crystallographic structures and Bragg data, PDF analysis shows that significant and systematic local distortions are present. As such, the average structures give a poor fit to PDF data for all samples except substitution- and vacancy-free LaFeO_3_. Some improvement in fit is possible using small-box fitting of models based on structures containing mixed BO*_n_* coordination polyhedra such as Sr_8_Fe_8_O_23_, Sr_4_Fe_4_O_11_, and Sr_2_Fe_2_O_5_.

To gain better local structural insight, we report big-box analyses that produce models consistent with both neutron and X-ray PDFs, Bragg data, and local chemical expectations. This approach is tested using LaFeO_3_, where it successfully reproduces both the polyhedral tilts of the *Pnma* structure and the expected patterns of atomic displacements from thermal motion. Big-box fitting of the oxidized samples shows a systematic increase in local disorder from LaFeO_3_ to La_0.5_Sr_0.5_FeO_3_ to La_0.6_Sr_0.4_FeO_3_ to La_0.6_Sr_0.4_Fe_0.67_Mn_0.33_O_3_. Data-derived bond distance and bond valence sum distributions suggest the predominance of Mn^4+^ in LSFM, consistent with a limiting composition of La_0.6_Sr_0.4_Fe^3+^_0.6_Fe^4+^_0.07_Mn^4+^_0.33_O_3_. We note, however, that oxidation states in a real material will depend strongly on the homogeneity of La and Sr substitutions on both a short- and long-range scale. For the reduced samples, PDF analysis suggests the predominance of square-pyramidal BO_5_ defects, consistent with related systems.^69^ These are accompanied by significant local distortions of neighboring BO_6_ octahedra leading to high-*r* shoulders on the B–O distance distributions. Distributions of local coordination polyhedra are again consistent with a systematic increase in local disorder from La_0.5_Sr_0.5_FeO_2.75_ to La_0.6_Sr_0.4_FeO_2.8_ to La_0.6_Sr_0.4_Fe_0.67_Mn_0.33_O_2.8_.

Overall, the systematic study of a series of well-characterized compositions alongside key reference materials gives significant insight into the local structures that underpin the function of these technologically important materials. Good agreement between the Rietveld-extracted oxygen content and that derived chemically is important for calibrating *operando* neutron diffraction experiments on working chemical looping reactors, while the local structural knowledge will inform the design of next-generation oxygen carrier materials.

## Supporting Information Available

Magnetic moment as a function of temperature from Rietveld analysis of variable-temperature neutron diffraction data for La_0.6_Sr_0.4_FeO_3−δ_ and La_0.6_Sr_0.4_Fe_0.67_Mn_0.33_O_3−δ_. Plot of key structural parameters from big-box PDF models (PDF)
CIF files for all Rietveld refinements (ZIP)
