# Supplementary material for: Average and Local Structure of La1–xSrxFe1–yMnyO3−δ Chemical Looping Oxygen Carrier Materials
Source: Chem Mater. 2025 May 2;37(9):3471–82. doi: 10.1021/acs.chemmater.5c00388 (PMC12079801; doi:10.1021/acs.chemmater.5c00388)

Supplementary Information: Average and local structure of $La_{1-x}Sr_xFe_{1-y}Mn_yO_{3-\delta}$ chemical looping oxygen carrier materials

Daniel M. Telford,[†] Wenting Hu,[†] Ian S. Metcalfe,[†] Martin O. Jones,[‡] Paul F. Henry,[‡] John S. O. Evans.[§,*]

†School of Engineering, Newcastle University, Newcastle-upon-Tyne, U.K.

‡ISIS Neutron and Muon Source, Science and Technology Facilities Council, Rutherford Appleton Laboratory, Didcot, U.K.

§Department of Chemistry, Durham University, Durham, U.K.

Rietveld Refinements

Results of the Rietveld refinements described in Table 1 of the main text have been deposited as powder CIF files. These can be viewed, for example, using pdCIFplotter by Matthew R. Rowles (https://doi.org/10.1107/S1600576722003478).

Sample ID and/or target composition	Space group	R_{wp} (%)	CIF Filename
1. $LaFeO_3$	62.448	2.23	1_pol126522_LaFeO3_pdcif.cif
2. LSF641 $La_{0.6}Sr_{0.4}FeO_3$	167.103	2.76	2c_pol126523_LSF641_pdcif.cif
3. LSF551 $La_{0.5}Sr_{0.5}FeO_3$	$R\bar{3}c$	2.91	3c_pol126525_LSF551_pdcif.cif
4. LSFM $La_{0.6}Sr_{0.4}Fe_{0.67}Mn_{0.33}O_3$	167.103	3.88	4c_pol126524_LSFM_pdcif.cif
5. Reduced LSF641 $La_{0.6}Sr_{0.4}FeO_{2.8}$	$Pm\bar{3}m$ / 167.108	3.96	5d_pol126526_LSF641_reduced_pdcif.cif
6. Reduced LSF551 $La_{0.5}Sr_{0.5}FeO_{2.75}$	$Pm\bar{3}m$ / 167.108	3.69	6b_pol126528_LSF551_reduced_pdcif.cif
7. Reduced LSFM $La_{0.6}Sr_{0.4}Fe_{0.67}Mn_{0.33}O_{2.8}$	$Pm\bar{3}m$ / 167.108	3.74	7c_pol126527_LSFM_reduced_pdcif.cif
8. $SrFeO_{2.875}$	$I4/mmm$	2.75	8_pol126531_SrFeO3-d_pdcif.cif
9. $SrFeO_{2.75}$	$Cmmm$	3.63	9c_pol126530_SrFeO2.75_pdcif.cif
10. $SrFeO_{2.5}$	$Ima2$ / 30.122	3.26	10b_pol126529_SrFeO2.5_pdcif.cif

Magnetic moments of LSF641 and LSFM from neutron diffraction

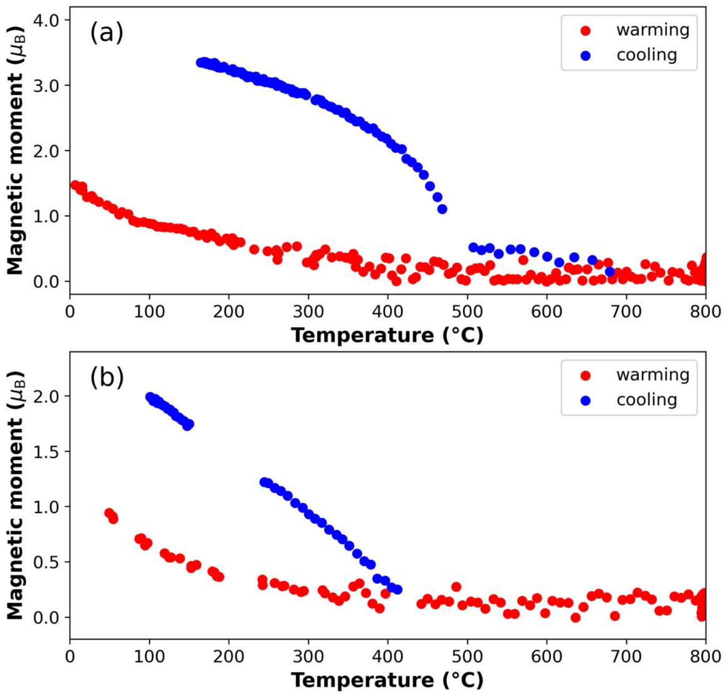

Figure S1. Magnetic moments extracted from Rietveld fitting of POLARIS powder diffraction data on warming and cooling samples of (a) LSF641 $La_{0.6}Sr_{0.4}FeO_{3-\delta}$ and (b) LSFM $La_{0.6}Sr_{0.4}Fe_{0.67}Mn_{0.33}O_{3-\delta}$. Data on warming are for an oxidised sample ($\delta \approx 0$) under Ar, conditions where O loss is low below T_N. Data on cooling are for samples reduced under the chemical looping conditions described in the text with $\delta \approx 0.2$. Warming data were collected in heat/hold-and-collect time slices such that the samples were heated at an overall constant rate. Gaps in the data are periods when the neutron beam was unavailable. For LSF the sample was warmed over 4 hours with data collected in 1-minute time slices. For LSFM the sample was warmed over 6 hours with data collected in 4-minute time slices. Cooling data was recorded at the natural cooling rate of the furnace. For LSF641 the cooling data were recorded over 2.5 hours, and for LSFM over 3.5 hours.

Big box PDF fits

The following figures show the results of big-box fitting using the method discussed in the main text. The table contains the following information for each model: unit-cell parameters, volume relative to a perovskite cubic unit cell (V_P), effective cell edge relative to a perovskite unit cell $a_P = (V_{cell}/V_P)^{1/3}$, number of atoms in the unit cell, breakdown of octahedral, square pyramidal and four coordinate BO_n polyhedra, number of O atoms and number of oxygen vacancies. As discussed in the main text, some models contained a single B site that was not 6, 5 or 3 coordinated; these are indicated by (+1).

ID	Formula	Name	Cell (Å)	V_P	a_P	Atoms	BO_6	$BO_{5/4}$	O	Vac	Model label
1	$LaFeO_3$	$LaFeO_3$	31.51717<br>31.48774<br>31.51717<br>90.036	512	3.930	2560	512	0	1536	0	lafeo3_08
2	$La_{0.6}Sr_{0.4}FeO_3$	LSF641	33.144<br>40.288	648	3.890	3240	648	0	1944	0	lsf641_ox_07
3	$La_{0.5}Sr_{0.5}FeO_3$	LSF551	33.079<br>40.247	648	3.893	3240	648	0	1944	0	lsf551_ox_09
4	$La_{0.6}Sr_{0.4}Fe_{0.67}Mn_{0.33}O_3$	LSFM	33.048<br>40.208	648	3.886	3240	648	0	1944	0	lsfm_ox_11
5	$La_{0.6}Sr_{0.4}FeO_{2.80}$	LSF641-RED	35.23	729	3.914	3500	441 (+1)	287	2042	145	lsf641_red_08
6	$La_{0.5}Sr_{0.5}FeO_{2.75}$<br>Square pyr Fe	LSF551-RED	35.2167	729	3.913	3463	367 (+1)	361	2005	182	lsf551_red_08c
6	$La_{0.5}Sr_{0.5}FeO_{2.75}$<br>Tetrahedral Fe	LSF551-RED	35.2169	729	3.913	3463	546Fe6 + 2Fe5	165Td + 16 sq pl	2005	182	lsf551_red_tet_07b
7	$La_{0.6}Sr_{0.4}Fe_{0.67}Mn_{0.33}O_{2.80}$<br>Mn 5 coord	LSFM-RED	35.218	729	3.913	3500	441Fe (+1)	44Fe + 243Mn	2042	145	lsfm_red_15c
7	$La_{0.6}Sr_{0.4}Fe_{0.67}Mn_{0.33}O_{2.80}$<br>Mn 6 coord	LSFM-RED	35.218	729	3.913	3500	198Fe + 243Mn (+1)	287Fe	2042	145	lsfm_red_16d
7	$La_{0.6}Sr_{0.4}Fe_{0.67}Mn_{0.33}O_{2.80}$<br>Mn mix	LSFM-RED	35.218	729	3.913	3500	294Fe + 147Mn (+1)	191Fe + 96Mn	2042	145	lsfm_red_27e

Figure S2. For each model reported, the figure panels contain:

(a) Histogram of B–O bond lengths. FeO_6 in brown, FeO_5 or FeO_4 in yellow, MnO_6 in green, MnO_4 in black and the sum in grey.

(b) Histogram of A–O bond lengths. La in green, Sr in blue, sum in grey.

(c) Histogram of O–B–O bond angles; colours as in (a).

(d) Histogram of BO_n polyhedral bond distortion indices as defined in main text. Colours as in (a).

(e) Bond valence sums for B site cations.

(f) Bond valence sums for A site cations.

(g) Histogram of shifts in fractional coordinates for B site cations.

(h) Histogram of shifts in fractional coordinates for A site cations.

(i) Histogram of shifts in fractional coordinates for O.

(j) Fit to Polaris bank 4 Bragg data, with magnetic contributions included based on the Rietveld model where applicable. Observed, calculated and difference data shown in blue, red and grey, respectively. Time of flight range plotted corresponds to a d-spacing range of 0.50 to 3.71 Å.

(k) Fit to neutron PDF data. Colours as in (j).

(l) Fit to X-ray PDF data (where applicable). Colours as in (j).

01. $LaFeO_3$ oxidised (lafeo3_08)

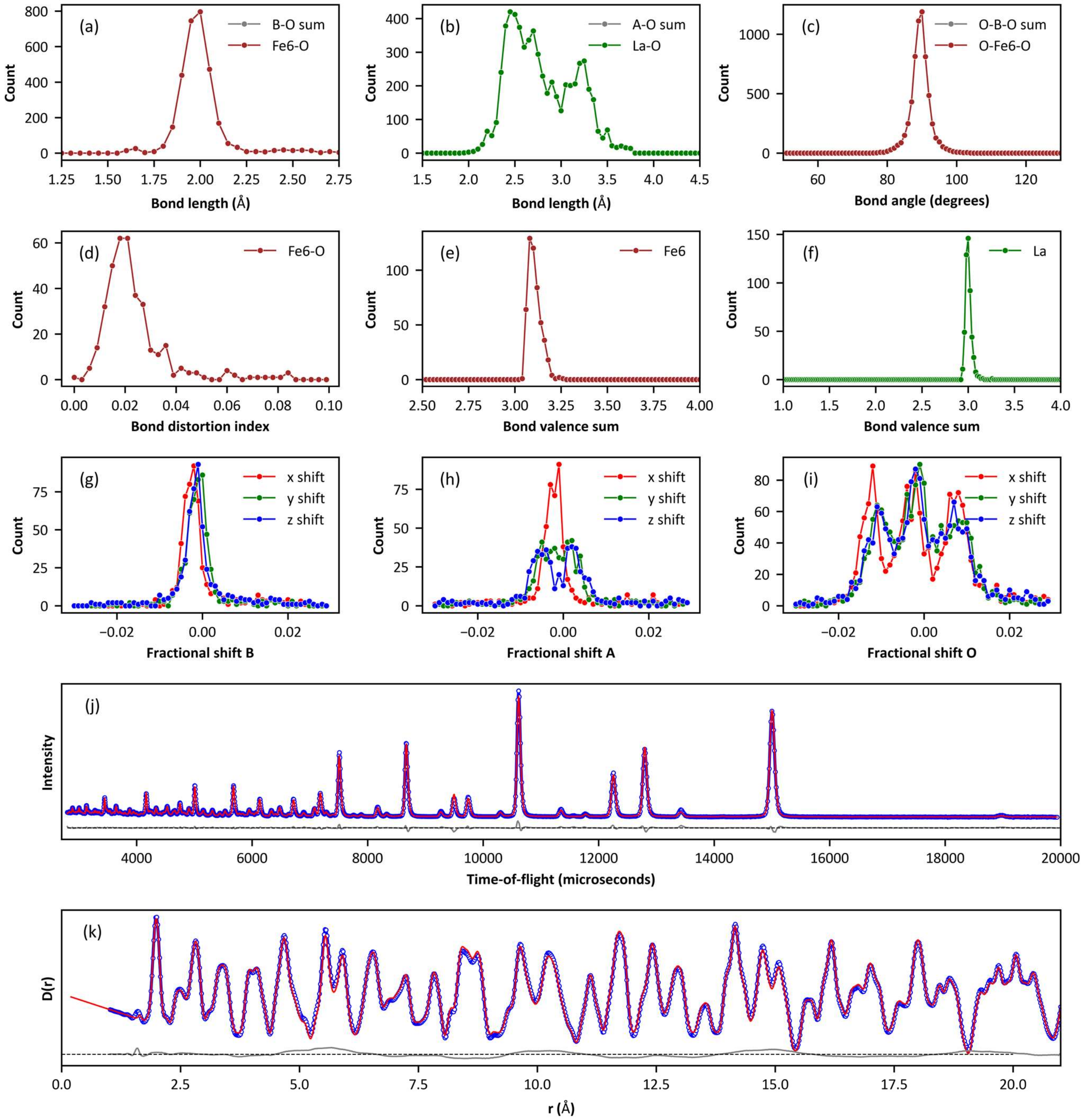

02. $La_{0.6}Sr_{0.4}FeO_3$ oxidised (lsf641_ox_07)

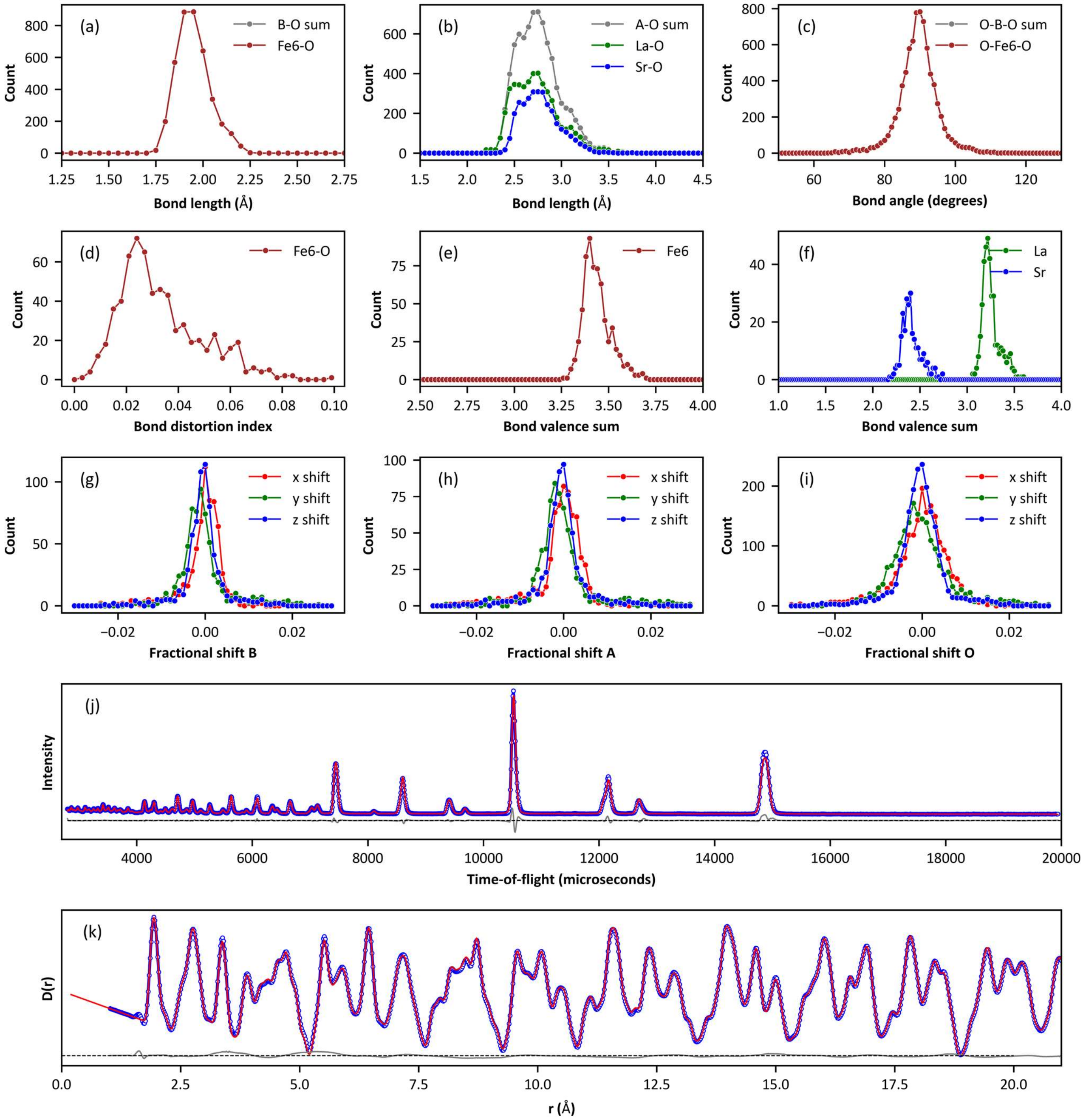

03. $La_{0.5}Sr_{0.5}FeO_3$ oxidised (lsf551_ox_09)

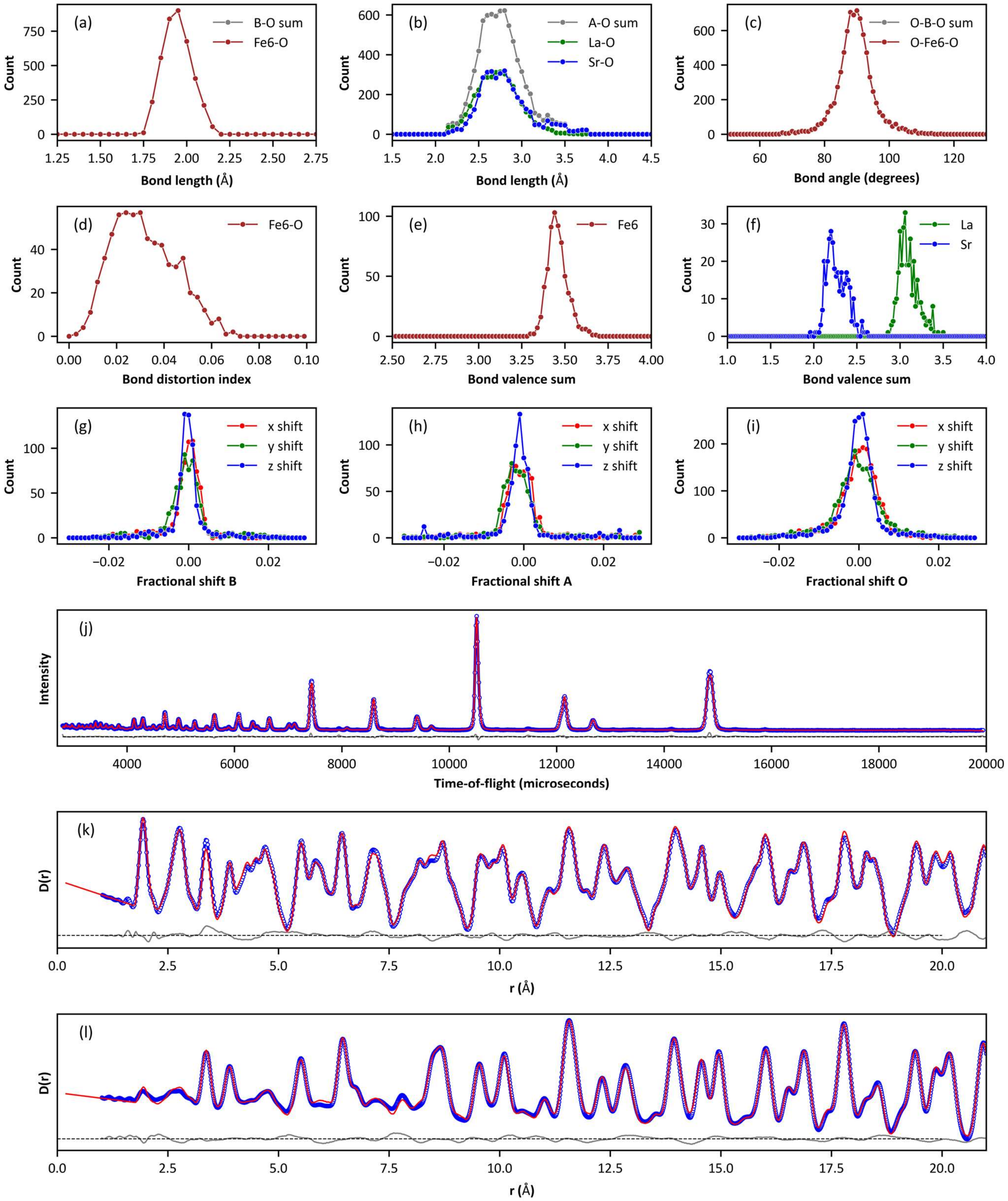

04. $La_{0.6}Sr_{0.4}Fe_{0.67}Mn_{0.33}O_3$ oxidised (lsfm_ox_11)

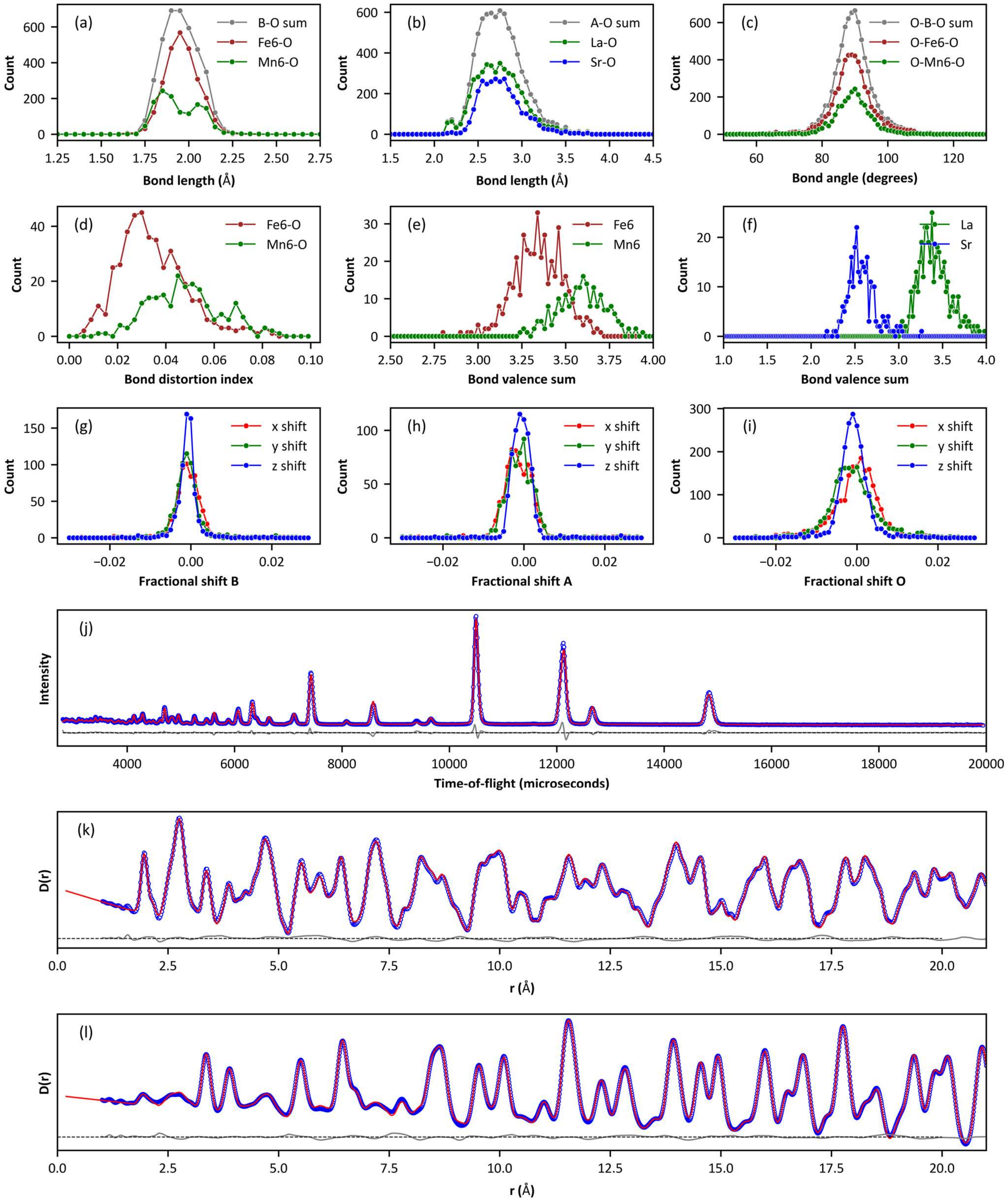

05. $La_{0.6}Sr_{0.4}FeO_{2.8}$ reduced (lsf641_red_08)

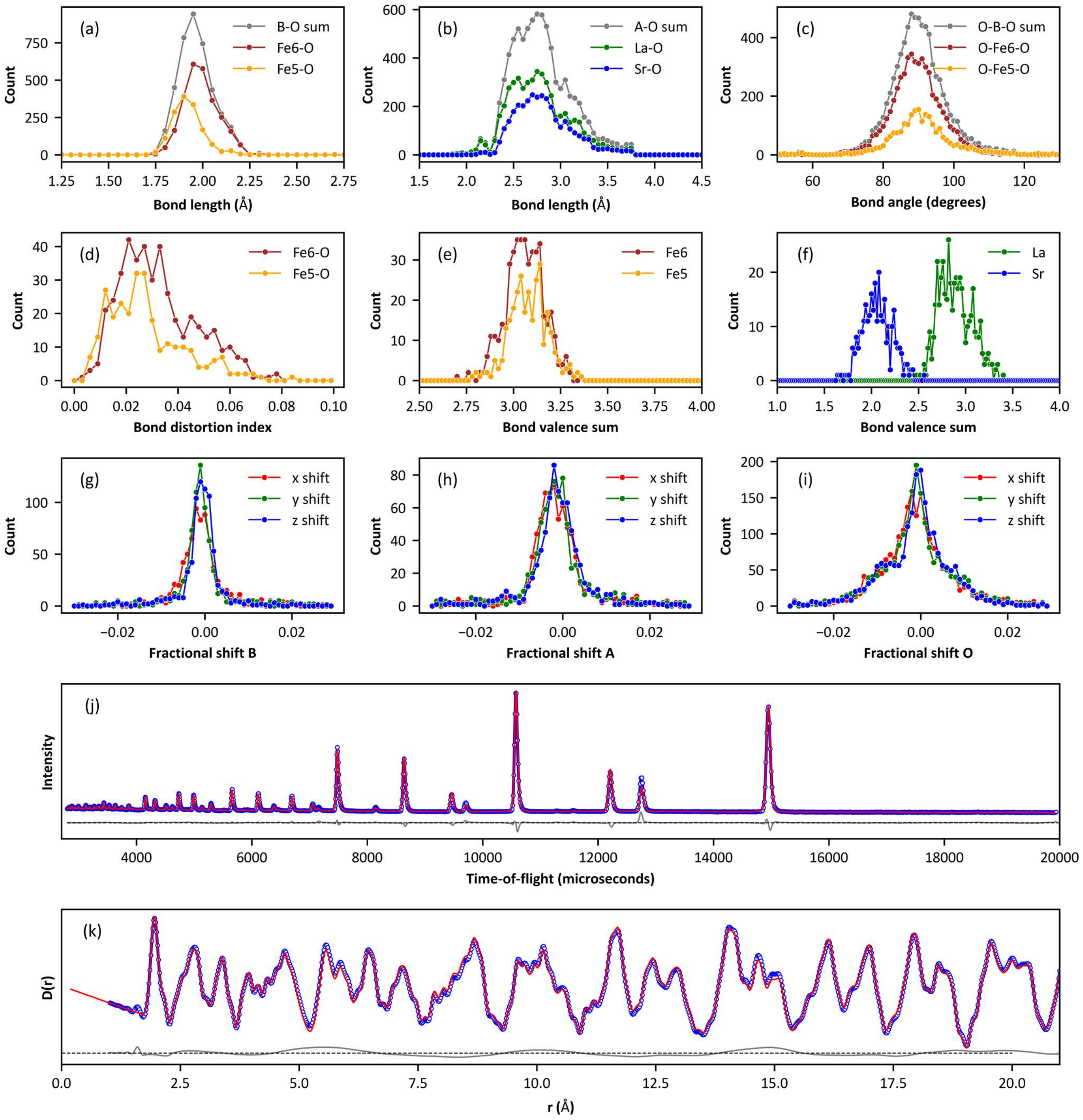

06. $La_{0.5}Sr_{0.5}FeO_{2.75}$ reduced FeO_5 square pyramids (lsf551_red_08c)

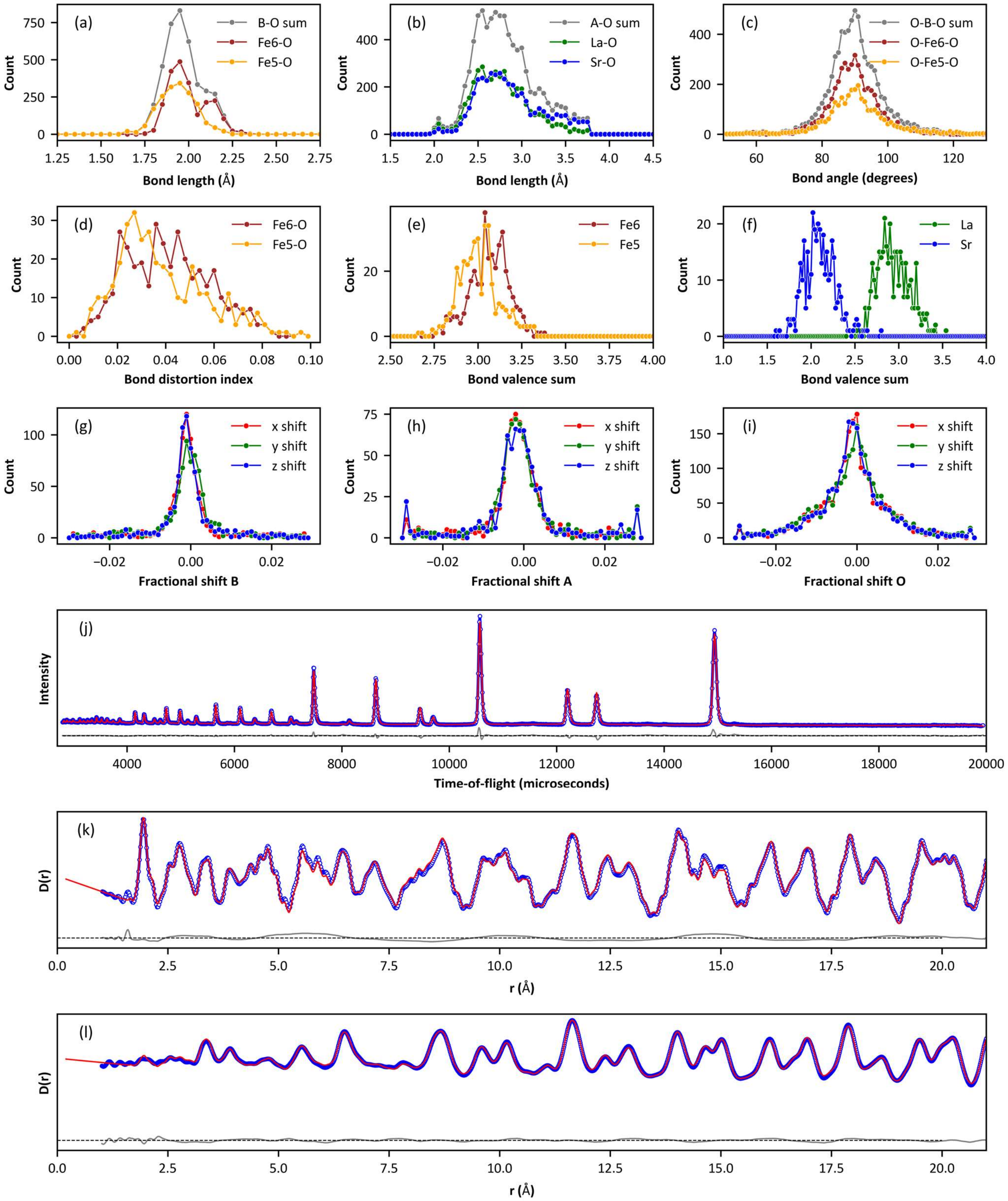

06. $La_{0.5}Sr_{0.5}FeO_{2.75}$ reduced FeO_4 tetrahedra (lsf551_red_tet_07b)

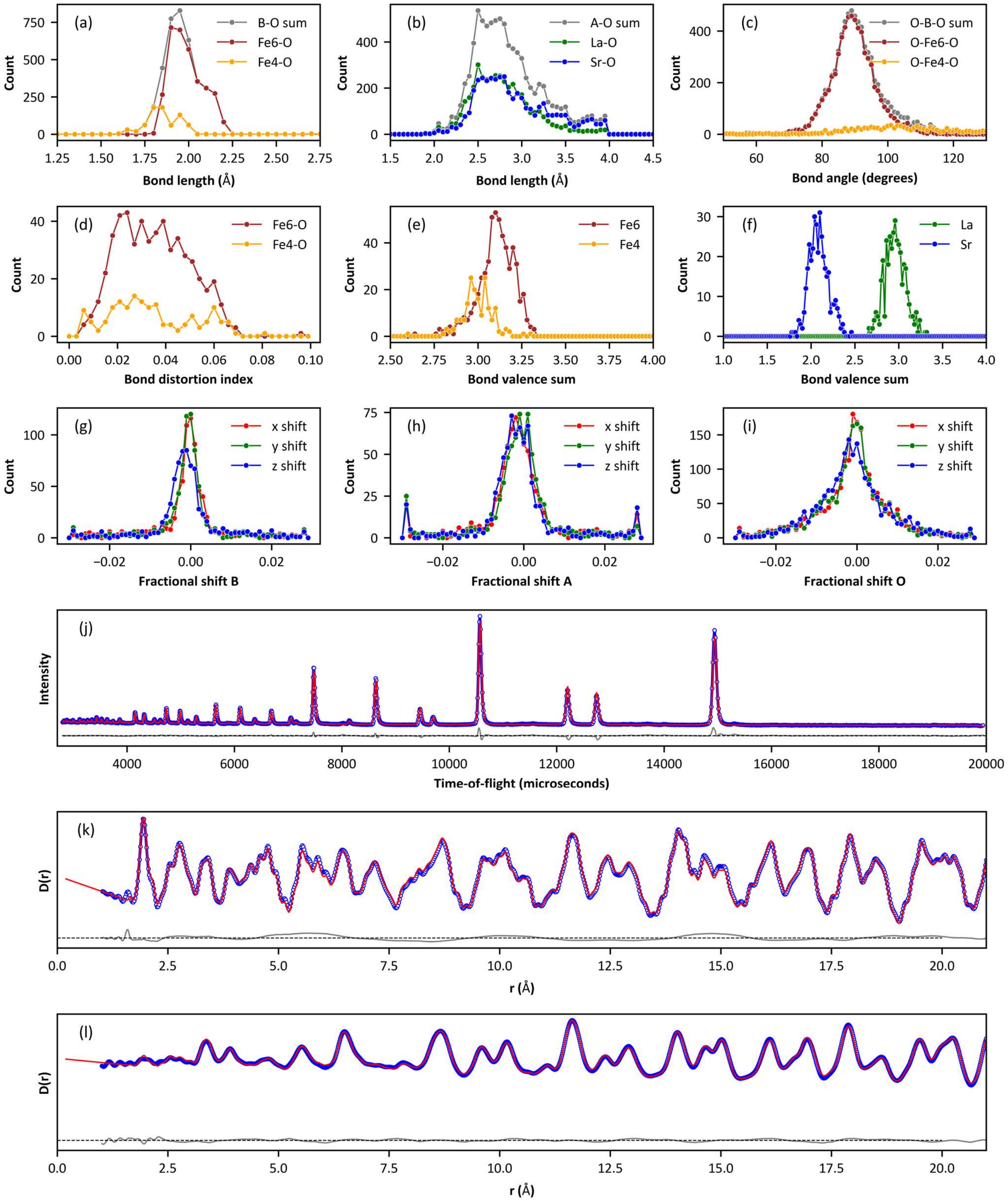

07. $La_{0.6}Sr_{0.4}Fe_{0.67}Mn_{0.33}O_{2.8}$ reduced FeO_6 (+ FeO_5) + MnO_5 (lsfm_red_15c)

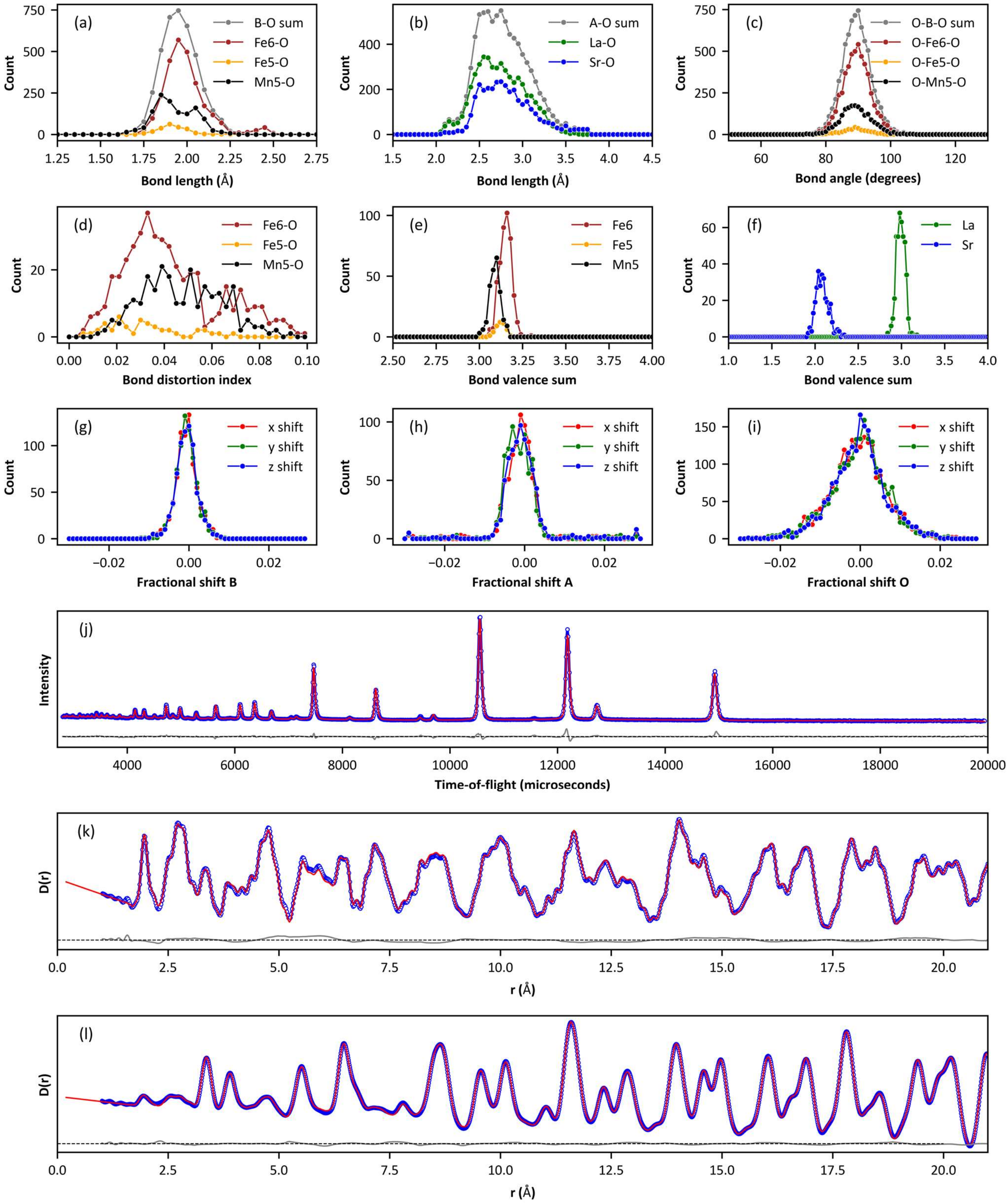

07. $La_{0.6}Sr_{0.4}Fe_{0.67}Mn_{0.33}O_{2.8}$ reduced FeO_6 + FeO_5 + MnO_6 (lsfm_red_16d)

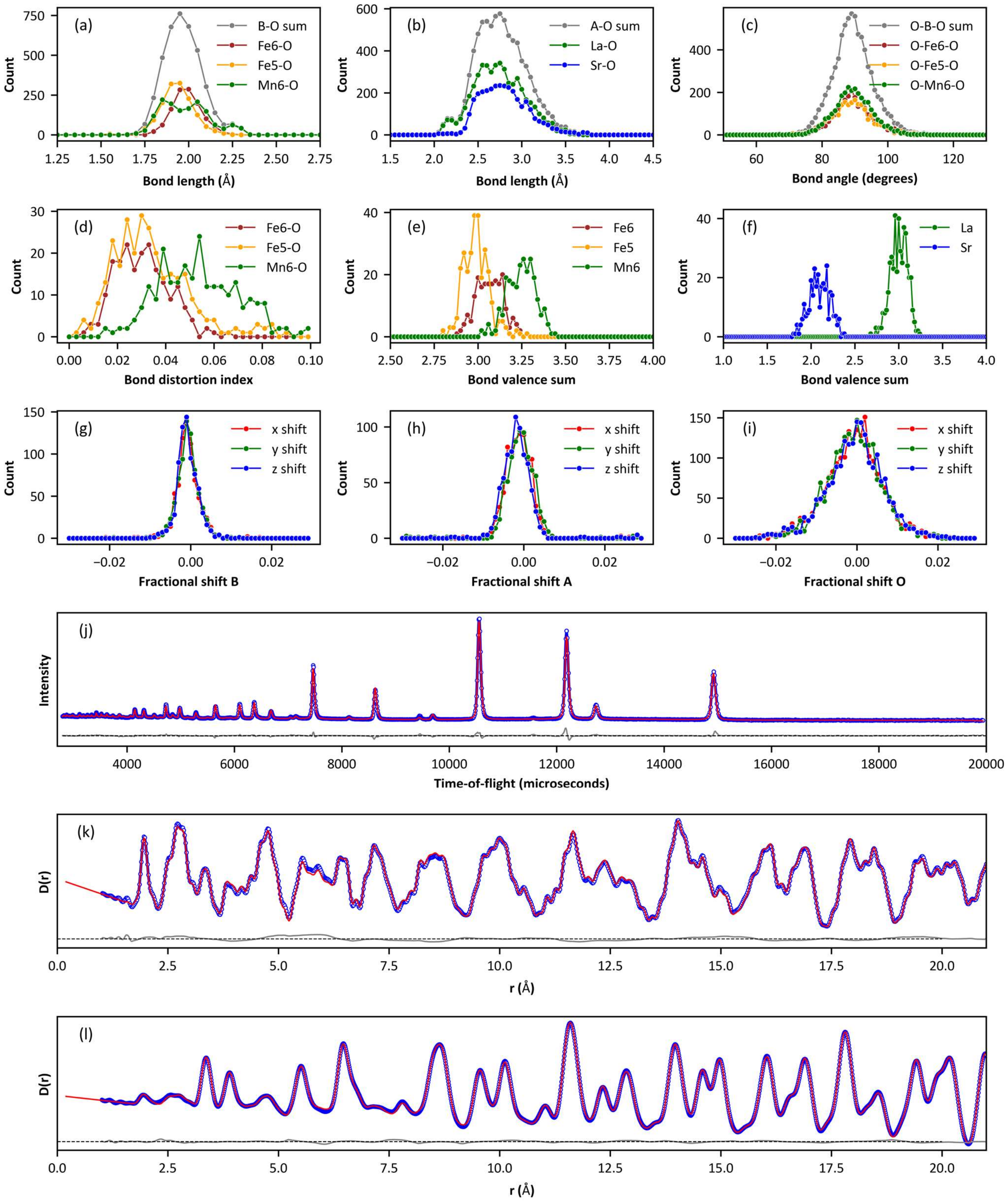

07. $La_{0.6}Sr_{0.4}Fe_{0.67}Mn_{0.33}O_{2.8}$ reduced FeO_6 + FeO_5 + MnO_6 + MnO_5 mixture (lsfm_red_27e)

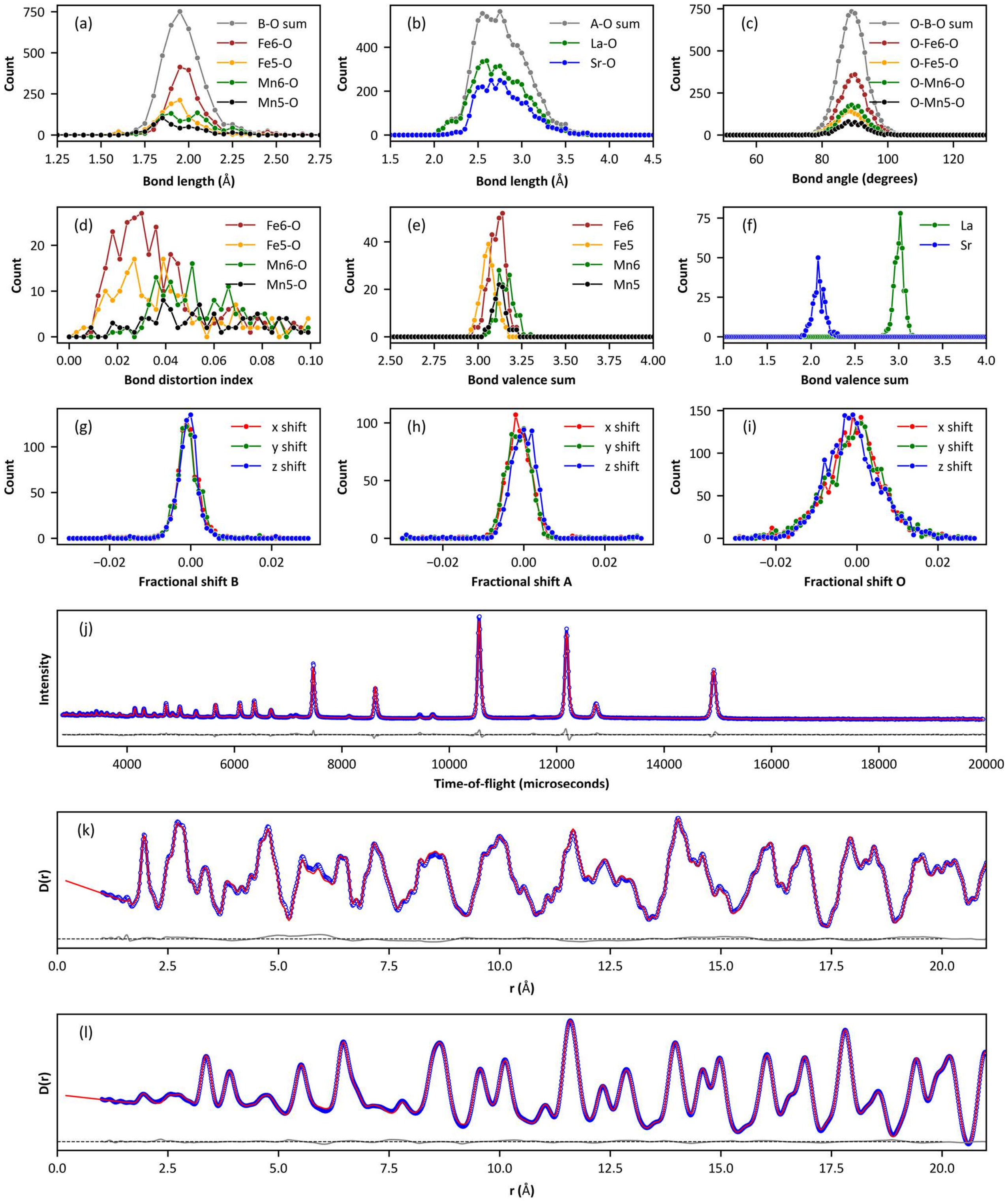

Supplement: Supplementary file 1 — cm5c00388_si_001.pdf [file cm5c00388_si_001.pdf]
